# MGA, L3MBTL2 and E2F6 determine genomic binding of the non-canonical Polycomb repressive complex PRC1.6

**DOI:** 10.1371/journal.pgen.1007193

**Published:** 2018-01-30

**Authors:** Bastian Stielow, Florian Finkernagel, Thorsten Stiewe, Andrea Nist, Guntram Suske

**Affiliations:** 1 Institute of Molecular Biology and Tumor Research (IMT), Philipps-University of Marburg, Marburg, Germany; 2 Genomics Core Facility, Center for Tumor Biology and Immunology (ZTI), Philipps-University of Marburg, Marburg, Germany; SPAIN

## Abstract

Diverse Polycomb repressive complexes 1 (PRC1) play essential roles in gene regulation, differentiation and development. Six major groups of PRC1 complexes that differ in their subunit composition have been identified in mammals. How the different PRC1 complexes are recruited to specific genomic sites is poorly understood. The Polycomb Ring finger protein PCGF6, the transcription factors MGA and E2F6, and the histone-binding protein L3MBTL2 are specific components of the non-canonical PRC1.6 complex. In this study, we have investigated their role in genomic targeting of PRC1.6. ChIP-seq analysis revealed colocalization of MGA, L3MBTL2, E2F6 and PCGF6 genome-wide. Ablation of MGA in a human cell line by CRISPR/Cas resulted in complete loss of PRC1.6 binding. Rescue experiments revealed that MGA recruits PRC1.6 to specific loci both by DNA binding-dependent and by DNA binding-independent mechanisms. Depletion of L3MBTL2 and E2F6 but not of PCGF6 resulted in differential, locus-specific loss of PRC1.6 binding illustrating that different subunits mediate PRC1.6 loading to distinct sets of promoters. Mga, L3mbtl2 and Pcgf6 colocalize also in mouse embryonic stem cells, where PRC1.6 has been linked to repression of germ cell-related genes. Our findings unveil strikingly different genomic recruitment mechanisms of the non-canonical PRC1.6 complex, which specify its cell type- and context-specific regulatory functions.

## Author summary

Polycomb group proteins assemble in two major repressive multi-subunit complexes (PRC1 and PRC2), which play important roles in many physiological processes, including stem cell maintenance, differentiation, cell cycle control and cancer. In mammals, six different groups of PRC1 complexes exist (PRC1.1 to PRC1.6), which differ in their subunit composition. The mechanisms that target the different PRC1 complexes to specific genomic sites appear diverse and are poorly understood. In this study, we have investigated the genomic targeting mechanisms of the non-canonical PRC1.6 complex. In PRC1.6, the defining subunit PCGF6 is specifically associated with several proteins including the transcription factors MGA and E2F6, and the histone-binding protein L3MBTL2. We found that MGA is absolutely essential for targeting PRC1.6. MGA executes recruitment of PRC1.6 to its target sites through two distinct functions. On the one hand it acts as a sequence-specific DNA-binding factor; on the other hand it has a scaffolding function, which is independent of its DNA binding capacity. E2F6 and L3MBTL2 are also important in genomic targeting of PRC1.6 as they promote binding of PRC1.6 to different sets of genes associated with distinct functions. Our finding that different components specify loading of PRC1.6 to distinct sets of genes could establish a paradigm for other chromatin-associated complexes.

## Introduction

Polycomb group (PcG) protein complexes play crucial roles in many physiological processes, including stem cell maintenance, differentiation, cell cycle control and cancer [1–4]. PcG complexes repress transcription through various mechanisms including changes in histone modification, polynucleosome compaction and direct interaction with the transcription machinery [1,5]. Two major complexes exist in mammals, the Polycomb repressive complexes 1 and 2 (PRC1 and PRC2), which differ in their enzymatic activity. PRC1 contains the E3 ligase RING1/2, which catalyzes ubiquitination of histone H2A at lysine 119 (H2AK119ub1), while PRC2 contains the methyltransferase EZH2 (Enhancer of Zeste Homolog 2) that catalyzes tri-methylation of histone H3 (H3K27me3). It has long been considered that H3K27me3 is required for PRC1 binding to chromatin. However, this view was challenged when it was found that a number of PRC1 complexes exist, which lack H3K27me3-binding CBX (Chromo Box) subunits [6–9].

Six major PRC1 complexes have been described and each contains a defining PCGF (Polycomb Group Ring Finger) subunit (PCGF1-6), the RING1/2 E3 ubiquitin ligase, RYBP/YAF2 (RING1 and YY1 binding protein/YY1 Associated Factor 2) or a CBX protein, and a unique set of associated proteins [7,8]. The canonical PCR1 complexes are PRC1.2, which contains PCGF2 (MEL-18), and PRC1.4, which contains PCGF4 (BMI1). They are recruited to chromatin by the H3K27me3 mark deposited by PRC2. By contrast, the non-canonical (ncPCR1s) PRC1.1, PRC1.3, PRC1.5 and PRC1.6 are targeted to chromatin by H3K27me3-independent mechanisms. Significantly, ncPRC1s are responsible for H2A ubiquitination (H2AK119ub1), which leads to recruitment of PRC2 and downstream H3K27me3 deposition [10].

Our knowledge of the targeting of ncPRC1 complexes to their genomic sites is limited. The PCGF1-containing PRC1.1 variant is recruited to non-methylated CpG islands via the histone methyltransferases Kdm2b (Lysine (K)-specific demethylase 2b), which binds to non-methylated CpG islands [6,9]. Recruitment of the PCGF3/5-containing ncPRC1s to the inactive X-chromosome is mediated by the Xist-RNA [11].

**Fig 1:**
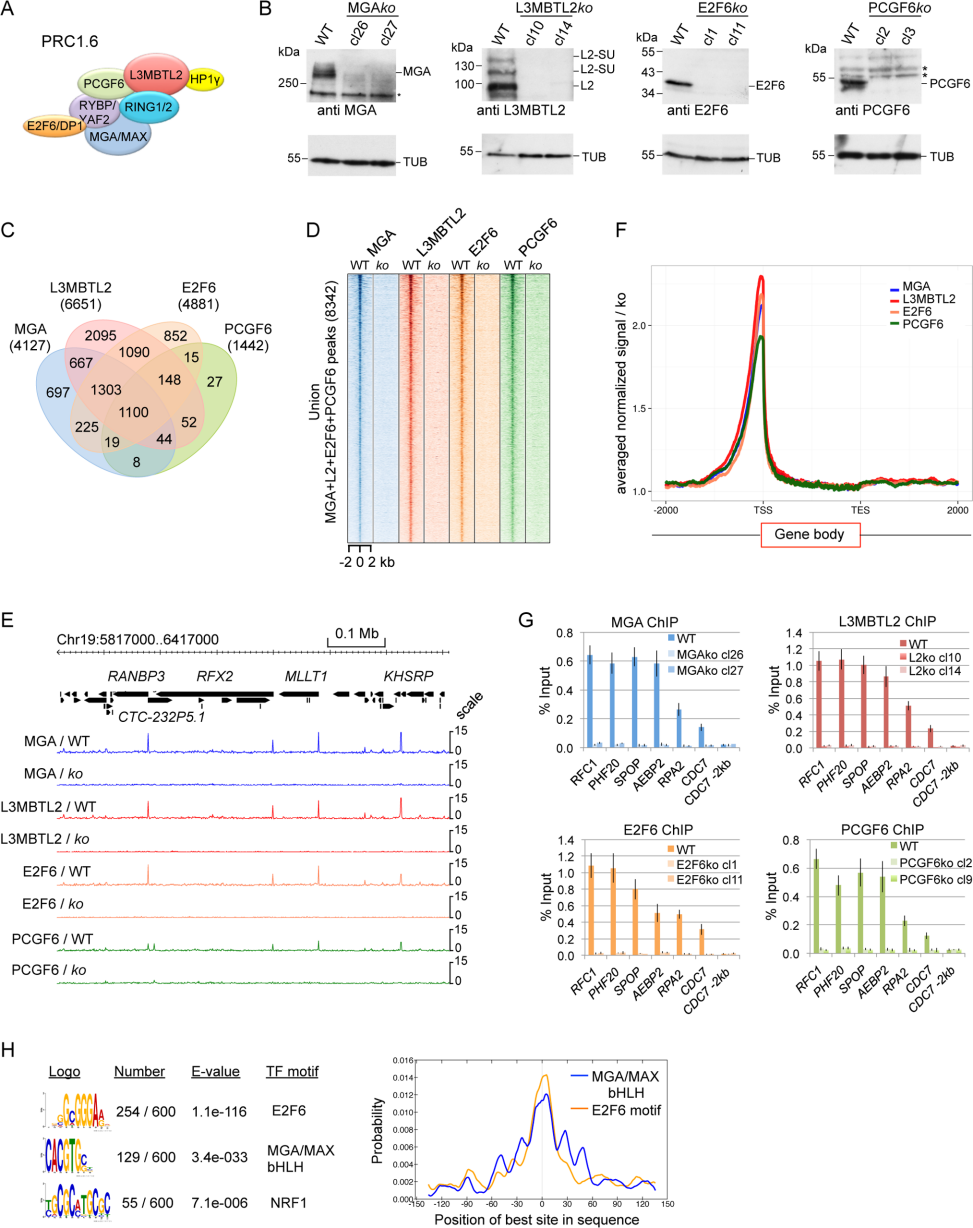
MGA, L3MBTL2, E2F6 and PCGF6 colocalize in 293 cells. (A) Schematic representation of PRC1.6 core components. (B) Western blot analysis of MGA, L3MBTL2, E2F6 and PCGF6 expression in wild type 293 cells (wt) and in corresponding MGA-, L3MBTL2-, E2F6- and PCGF6-depleted cell clones (MGA*ko*, L3MBTL2*ko*, E2F6*ko* and PCGF6*ko*). Re-probing for tubulin (TUB) controlled loading of extracts. (C) Venn diagrams showing the overlap of MGA, L3MBTL2, E2F6 and PCGF6 binding regions in HEK293 cells. The total number of high-confidence MGA, L3MBTL2, E2F6 and PCGF6 ChIP-seq peaks (≥30 tags, ≥3-fold enrichment over knockout control) and their overlap is shown. (D) A heat map view of the distribution of union MGA, L3MBTL2, E2F6 and PCGF6 peaks in HEK293 cells (n = 8342) at +/- 2 kb regions centred over the MGA peaks. (E) Representative genome browser screenshots of a 0.7 Mb region of chromosome 19 showing co-localization of MGA, L3MBTL2, E2F6 and PCGF6 at the *CTC-232P5*.*1*, *RFX2*, *MLLT1* and *KHSRP* promoters. (F) Distribution of MGA, L3MBTL2, E2F6 and PCGF6 peaks relative to positions -2000 bp upstream to +2000 bp downstream of gene bodies. TSS, transcription start site; TES, transcription end site. (G) ChIP-qPCR analysis of MGA, L3MBTL2, E2F6 and PCGF6 binding to selected promoters. The region -2 kb upstream of the *CDC7* promoter served as a negative control. Percent of input values represent the mean of at least three independent experiments +/- SD. (H) Sequence motifs enriched in PRC1.6 binding regions. Logos were obtained by running MEME-ChIP with 300 bp summits of the top 600 union MGA-L3MBTL2-E2F6-PCGF6 ChIP-seq peaks. The numbers next to the logos indicate the occurrence of the motifs, the statistical significance (E-value) and the transcription factors that bind to the motif. Right panel, local motif enrichment analysis (CentriMo) showing central enrichment of the MGA/MAX bHLH and the E2F6/DP1 binding motifs within the 300 bp peak regions. The NRF1 binding motif was not centrally enriched.

The subunit composition of the different ncPCR1s is specific and potentially revealing. While PRC1.6 (also known as E2F6-PRC1 and PCGF6-PRC1) is similar if not identical to L3MBTL2 (Lethal(3)Malignant Brain Tumor-Like 2)-containing complexes [12,13] and the E2F6 repression complex [14], it is specifically associated with several proteins that are not found in other ncPRC1s (Fig 1A), [7,15]. MGA (MAX Gene-Associated protein, also abbreviated as MGAP by UniProt) contains two DNA-binding domains, a T-box domain and a bHLH (basic helix-loop-helix) domain. MGA interacts with MAX (Myc-associated Factor X), and E2F6 interacts with DP-1 or DP-2 (transcription factor DP-1 or DP-2). Heterodimeric MGA/MAX binds E-boxes, and heterodimeric E2F6/DP-1/2 binds to E2F recognition sequences *in vitro* [16–18]. L3MBTL2 contains four MBT domains that bind to mono- and di-methylated histone H3 and H4 tails *in vitro* [19–21]. Full-length L3MBTL2 can also interact with histones independent of their lysine methylation state [12,20]. The association of PRC1.6 with the sequence-specific DNA binding proteins MGA/MAX and E2F6/DP1, and with the histone-interacting protein L3MBTL2 suggests that these proteins could play a role in locus-specific recruitment of PRC1.6. Crucially, this notion has not been addressed experimentally.

In mouse embryonic stem cell (ESC) an essential role for the corresponding PRC1.6 subunits in specification and proliferation has been demonstrated. Mga and Pcgf6 were identified as essential self-renewal genes in ESCs by a genome-wide RNAi screen [22]. A more recent knockout study revealed that Mga is essential for survival of mouse pluripotent cells during peri-implantation development and for growth of ESC cultures [23]. L3mbtl2-deficient ESCs retain characteristics of pluripotent cells but are severely impaired in proliferation [13]. Finally, the defining subunit of PRC1.6, Pcgf6, is expressed at high levels in mouse ES cells, where it is required for ESC identity [24,25]. The mechanism by which this occurs remains controversial. Two reports suggested a repressive function of Pcgf6 on mesodermal-specific [24] and on endodermal lineage genes [26], while Yang et al. suggested an PRC1.6-independent direct activator function of Pcgf6 on core ESC regulators such as Oct4, Sox2 and Nanog [25].

Here we describe the targeting mechanism of PRC1.6, an exemplar of the non-canonical PRC1 class, by detailing the role of MGA, L3MBTL2, E2F6 and PCGF6 in genomic binding site selection. We show that MGA, L3MBTL2, E2F6 and PCGF6 colocalize genome-wide in the context of PRC1.6. Taking advantage of CRISPR/Cas-mediated genetic ablation in HEK293 cells, we demonstrate that MGA is absolutely essential for binding of PRC1.6. By expression of MGA mutants in MGA*ko* cells, we found that the *bona fide* T-box and bHLH DNA-binding domains of MGA mediate binding to a subset of loci but are dispensable for others. We further demonstrate that L2MBTL2 and E2F6 determine differential binding of PRC1.6 to distinct promoters. Finally, we demonstrate that Mga, L3mbtl2 and Pcgf6 colocalize also in mouse ESCs. In particular, we found enrichment at promoters of meiosis-and germ-line-specific genes that were shown to be de-repressed on Max-, L3mbtl2- or Pcgf6-depletion. Together, our findings unveil strikingly different genomic recruitment mechanisms for a non-canonical Polycomb repressive complex, which specify its cell type- and context-specific regulatory functions.

## Results

### Genomic colocalization of MGA, L3MBTL2, E2F6 and PCGF6 in HEK293 cells

To identify the genomic binding sites of PRC1.6 and to gain mechanistic insights into its targeting, we focused on the roles of MGA, L3MBTL2, E2F6 and PCGF6 as these factors are specific to PRC1.6 (Fig 1A) and were not found in other ncPRC1s. We established HEK293 cell clones in which each of these four proteins was depleted individually using the CRISPR/Cas9-sgRNA system (S1 Fig) as controls at key steps in the analysis. By Western blotting we confirmed successful depletion of MGA, L3MBTL2, E2F6 and PCGF6 in several clones (Fig 1B).

Next, we determined MGA, L3MBTL2, E2F6 and PCGF6 occupancy by ChIP-seq using chromatin of the corresponding knockout cell lines as a reference for peak selection. Thereby, we were able to remove a number of perfectly shaped false positive ChIP-seq signals (S2A Fig) from the classified lists of binding sites. We obtained different peak strengths and different numbers of peaks for the different factors (L3MBTL2 > E2F6 > MGA > PCGF6), possibly due to the different performance of the antibodies resulting in different ChIP efficiencies. Stringent filtering of uniquely mapped reads (≥30 tags and ≥3-fold enrichment over the corresponding knockout control) yielded lists of high-confidence binding sites for each factor.

Comparison of the MGA, L3MBTL2, E2F6 and PCGF6 data sets revealed a very high degree of overlap (Fig 1C and 1D) reflecting colocalization (Fig 1E). Consistent with the role of PRC1 in regulating gene expression, the large majority of these sites were located close to the 5´-end of annotated transcripts (Fig 1F). We also confirmed colocalization of MGA, L3MBTL2, E2F6 and PCGF6 to a set of selected target promoters by conventional ChIP-qPCR analysis (Fig 1G).

The overlap of the MGA, L3MBTL2, E2F6 and PCGF6 ChIP-seq peaks shown in Fig 1C also suggests the existence of some genomic sites bound by only one of the four factors. However, the majority of the potential factor-specific sites was removed when we compared filtered peaks with unfiltered MACS peaks (S2B–S2E Fig). Moreover, visual genome browser inspection of the remaining potential subunit-specific peaks indicated the shared presence of MGA, E2F6, L3MBTL2 and PCGF6 at all examined sites (S2B–S2E Fig). Hence, our ChIP-seq results indicate that all four factors bind to the same genomic loci *in vivo*. This conclusion is strongly supported by the complete absence of genomic L3MBTL2, E2F6 and PCGF6 binding events in MGA-depleted cells (see below).

A *de novo* sequence motif analysis of the top 600 ranked MGA, L3MBTL2, E2F6 and PCGF6 binding sites revealed centrally enriched motifs that match *in vitro* recognition sequences for MGA/MAX (the E-Box, CACGTG) [17] and for E2F6/DP1 (GCGGGAA) [18] (Fig 1H). The abundant occurrence of the E-box and the E2F6 binding motif indicated that both, MGA and E2F6, could be important for recruitment of PRC1.6 to its specific sites in chromatin.

### MGA plays a crucial role in genomic targeting of PRC1.6

**Fig 2:**
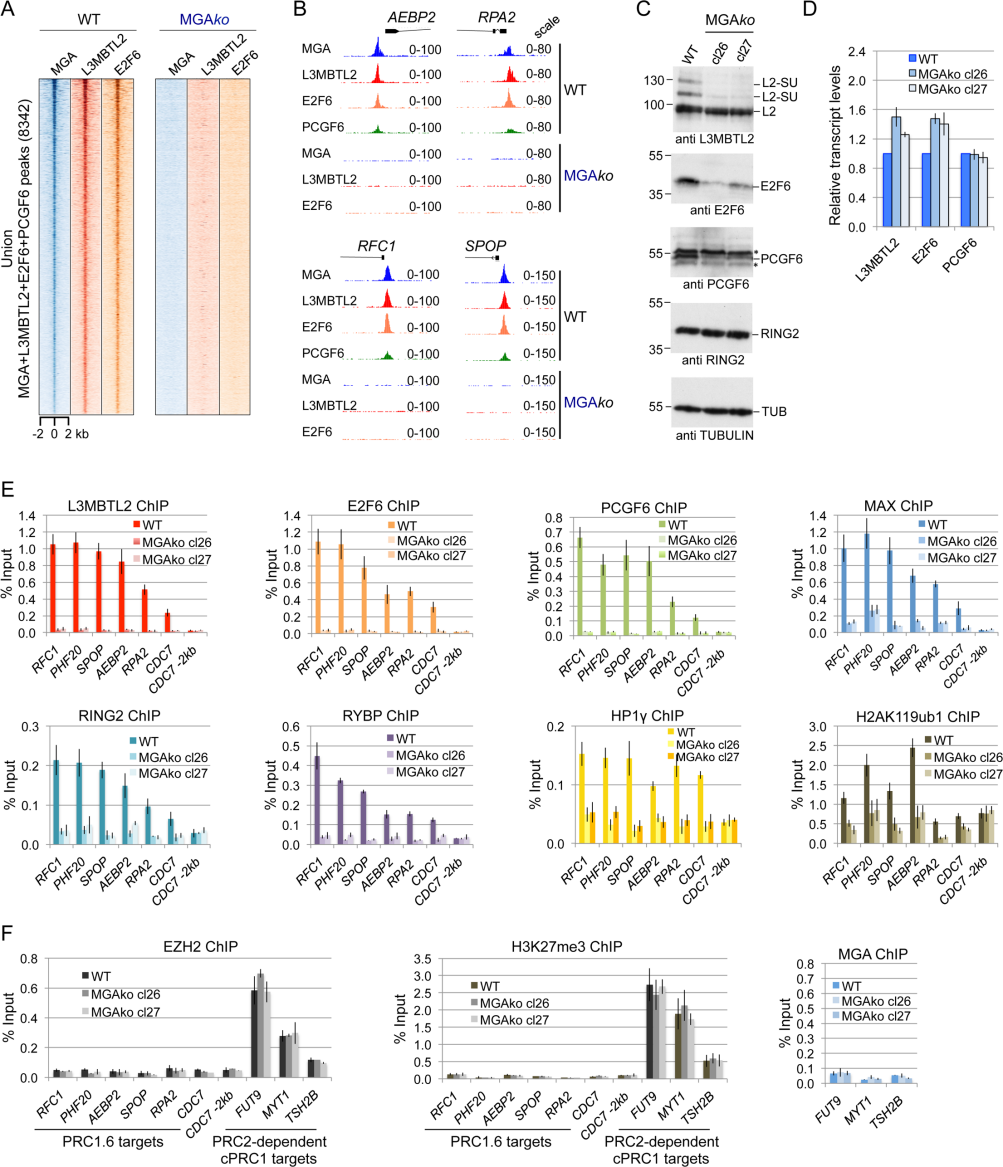
MGA is essential for genomic binding of PRC1.6. (A) Heat map view of the distribution of union MGA, L3MBTL2 and E2F6 peaks in wild type cells (n = 8342) and in MGA-depleted cells at +/- 2 kb regions centred over the MGA peaks. (B) Representative genome browser screenshots showing binding of MGA, L3MBTL2, E2F6 and PCGF6 to the *AEBP2*, *RPA2*, *RFC1* and *SPOP* promoters in wild type cells. MGA-depleted cells lack binding of L3MBTL2 and E2F6. (C) Western blot analysis of L3MBTL2, E2F6, PCGF6 and RING2 in wild type HEK293 cells and in two different MGA-depleted clones (cl26 and cl27). The anti-Tubulin blot served as a loading control. (D) *L3MBTL2*-, *E2F6*- and *PCGF6* transcripts were determined in wild type cells and in MGA-depleted cell clones by RT-qPCR analysis. *B2M* transcript levels were used to normalize the data across samples, and transcript levels in wild type cells were arbitrarily set to 1. Data represent the average of technical replicates ± SD. (E) ChIP-qPCR data showing lack of L3MBTL2, E2F6, PCGF6, MAX, RING2, RYBP and HP1γ binding to representative PRC1.6 target promoters in MGA*ko* cells, and diminished deposition of H2AK119ub1. The *CDC7* -2kb region served as a negative control region. Percent of input values represent the mean of at least three independent experiments +/- SD. (F) PRC1.6 target promoters are not bound by PRC2 and lack H3K27me3. Local levels of EZH2 and H3K27me3 at selected PRC1.6 target promoters in wild type (WT) and in MGA*ko* cells (clones cl26 and cl27) were determined by ChIP-qPCR analysis. Genomic regions known to be bound by canonical PRC1 (*FUT9*, *MYT1* and *TSH2B*) served as positive control regions. These regions were not bound by MGA (right panel). Percent of input values represent the mean of at least three independent experiments +/- SD.

MGA and E2F6 are sequence-specific DNA binding factors; and L3MBTL2 is a histone-interacting protein. Having found that they colocalize genome-wide, we set out to investigate their interdependence in genomic targeting of PRC1.6. At first we focused on the role of MGA and examined whether binding of other PRC1.6 subunits was affected in MGA-depleted cells. ChIP-seq analysis revealed that MGA*ko* cells lack genome-wide binding of both L3MBTL2 and E2F6 (Fig 2A and 2B) indicating that MGA is crucial for genomic targeting of L3MBTL2 and E2F6 and potentially for the entire PRC1.6 complex. This finding was particularly unexpected since the E2F6/DP2 heterodimer binds E-box motifs readily *in vitro* [18]. Western blot analysis revealed that MGA-depleted cells contained markedly less E2F6 as well as PCGF6 (Fig 2C). The reduced protein levels of E2F6 and PCGF6 in MGA*ko* cells were likely due to impaired protein stability, as the transcript levels of *E2F6* and *PCGF6* were not reduced in MGA*ko* cells (Fig 2D). The protein level of L3MBTL2 in MGA*ko* cells was similar as in wild type cells. However, the fraction of SUMO-modified L3MBTL2 [20] was strongly reduced (Fig 2C), which may indicate that SUMOylation of L3MBTL2 in wild type cells takes place at the level of chromatin. Finally, the level of RING2 protein was unchanged in MGA-deficient cells.

To exclude that the lack of any E2F6 and L3MBTL2 binding in MGA*ko* cells was the result of inefficient ChIPs, we also probed a panel of selected target promoters by ChIP-qPCR. These experiments validated the lack of genomic L3MBTL2 and E2F6 binding in two different MGA*ko* clones (Fig 2E). We also analyzed for the presence of other PRC1.6 components including PCGF6, MAX, RING2, RYBP, HP1γ and the H2AK119ub1 mark. All factors as well as the H2AK119ub1 mark were present at the MGA target sites in wild type cells but were absent or, in the case of the H2AK119ub1 mark, markedly reduced in both MGA-depleted cell clones (Fig 2E). The global H2AK119ub1 levels were similar in wild type, MGA*ko*, L3MBTL2*ko*, E2F6*ko*, and PCGF6*ko* cells (S3 Fig) showing that the observed reduction of the H2AK119ub1 mark at the PRC1.6 target regions is due to changes in local RING2 deposition. Collectively, these results demonstrate that MGA is absolutely crucial for genomic loading of the entire PRC1.6 complex. Importantly, these results also indicate that E2F6, L3MBTL2 and PCGF6 bind to their genomic sites exclusively in the context of the PRC1.6 complex and are not recruited to chromatin independently of PRC1.6.

Since previous studies reported that H2AK119ub1 plays a critical role for recruitment of PRC2 followed by downstream deposition of H3K27me3 [10], we also tested for the presence of the catalytic PRC2 component EZH2 and for H3K27me3 (Fig 2F). Neither EZH2 nor H3K27me3 were enriched at the selected PRC1.6 loci suggesting that PRC1.6 binding is not generally interconnected with PRC2 binding. Importantly, we found considerable enrichment of EZH2 and H3K27me3 at known PRC2-dependent canonical PRC1 target sites. These canonical PRC1 binding sites were not bound by MGA, and the levels of EZH2 and H3K27me3 at these sites remained unchanged in MGA*ko* cells (Fig 2F). The absence of MGA at canonical PRC1 binding regions is consistent with genome-wide data that revealed only a low level of overlap between PCGF6 and other PCGFs in HEK293 cells [7].

### MGA promotes the genomic localisation of PRC1.6 through different mechanisms

**Fig 3:**
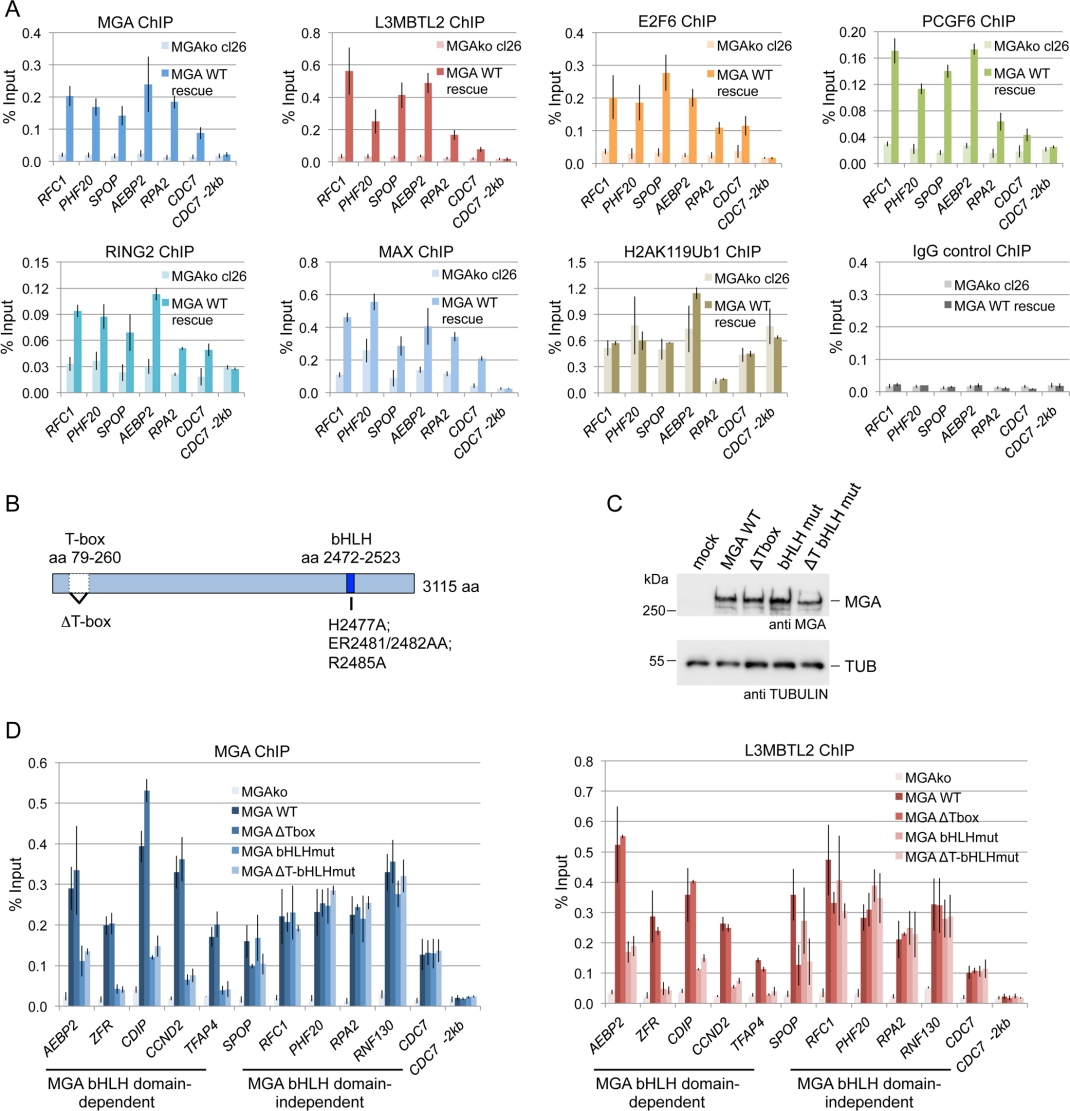
MGA promotes binding of PRC1.6 by DNA-binding-dependent and DNA-binding-independent mechanisms. (A) Expression of wild type MGA in MGA*ko* cells rescues binding of PRC1.6. ChIP-qPCR data showing binding of transiently expressed MGA and of endogenous L3MBTL2, E2F6, PCGF6, RING2 and MAX to representative PRC1.6 target promoters. The level of the H2AK119ub1 was not affected. Percent of input values represent the mean of at least three independent experiments +/- SD. (B) Schematic representation of the MGA ΔTbox and bHLH mutants. (C) Western blot analysis of wild type MGA and of the DNA-binding-deficient MGA mutants (ΔT-Box, bHLHmut and ΔTbHLHmut) expressed in MGA*ko* cells. The anti-Tubulin blot served as a loading control. (D) ChIP-qPCR analyses of MGA and L3MBTL2 binding to selected PRC1.6 target promoters in MGA*ko* cells and in MGA*ko* cells re-expressing wild type MGA (MGA WT) or DNA-binding-deficient MGA mutants (MGA ΔT-Box, MGA bHLHmut or MGA ΔTbHLHmut). The error bars denote SD; n = 3.

Given that MGA is essential for targeting of PRC1.6, it would be expected that re-expression of MGA would restore not only genomic binding of MGA but also of the other PRC1.6 components. To test this prediction, we expressed full-length MGA in MGA*ko* cells, and subsequently analyzed a panel of PRC1.6 target promoters for binding of exogenous MGA and of endogenous L3MBTL2, E2F6, PCGF6, MAX and RING2. Indeed, re-expression of MGA in MGA*ko* cells not only restored specific binding of MGA but also of the other PRC1.6 subunits (Fig 3A). We did not observe an increase of H2AK119ub1 levels at these promoters. Potentially, the short time span of transient MGA expression was not sufficient for the H2AK119ub1 mark to be deposited efficiently.

MGA contains two different DNA binding domains, a T-box domain close to the N-terminus and a bHLH domain in its C-terminal part (Fig 3B). To test whether these DNA binding domains account for genomic loading of PRC1.6, we generated two different types of DNA binding-deficient MGA mutants by deleting the entire T-box domain (MGA-ΔT, aa 79 –aa 264 deleted), and by replacing several critical amino acids in the bHLH domain [27,28] by alanine residues (MGA-bHLHmut, MGA-H2477A_ER2481/2482AA_R2485A). Compared with wild type MGA, binding of the MGA-bHLH mutant to several target promoters (*AEBP2*, *ZFR*, *CDIP*, *CCND2* and *TFAP4*) was strongly reduced (Fig 3D). We also observed reduced binding of the MGA-T-box deletion mutant to the *SPOP* promoter. However, both MGA mutants still bound to the *RFC1*, *PHF20*, *RPA2*, *RNF130* and *CDC7* promoters as efficiently as wild type MGA (Fig 3D). We also tested binding of an MGA double mutant in which both DNA binding domains were mutated simultaneously (MGA-ΔT-bHLHmut). Remarkably, the MGA-ΔT-bHLHmut double mutant still bound to these promoters as efficiently as wild type MGA. Importantly, the DNA binding-deficient MGA mutants also rescued binding of endogenous L3MBTL2 to the *RFC1*, *PHF20*, *RPA2*, *RNF130* and *CDC7* promoters but not to the MGA-bHLH-dependent *AEBP2*, *ZFR*, *CDIP*, *CCND2* and *TFAP4* promoters and to the MGA-T-Box-dependent S*POP* promoter (Fig 3D, right panel). These results suggest that MGA can recruit PRC1.6 to specific target sites by DNA-binding-dependent and by DNA-binding-independent mechanisms.

### L3MBTL2 and E2F6 contribute to genomic binding of PRC1.6

**Fig 4:**
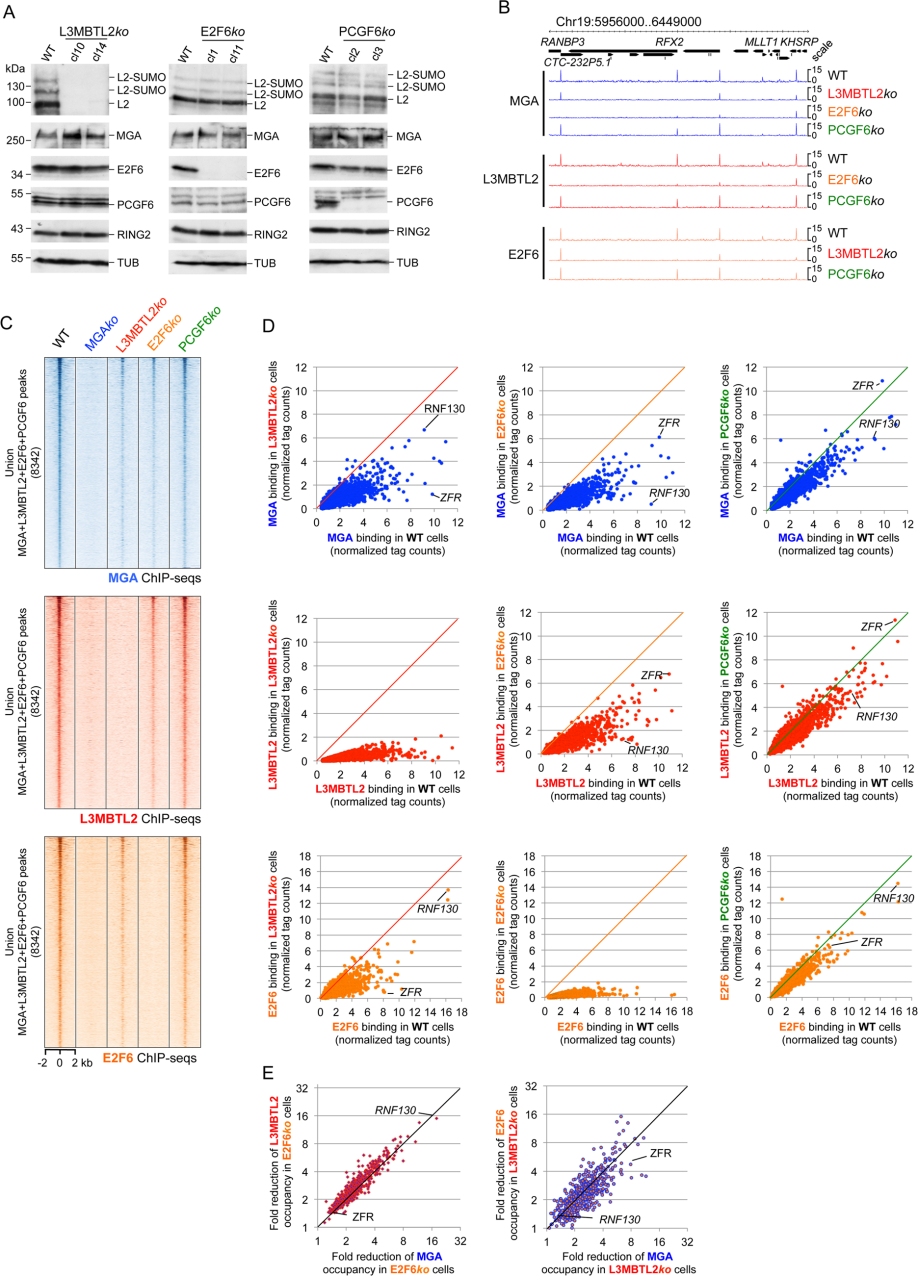
L3MBTL2 and E2F6 contribute to chromatin binding of PRC1.6. (A) Western blot analysis of L3MBTL2, MGA, E2F6, PCGF6 and RING2 in L3MBTL2*ko*, E2F6*ko* and PCGF6*ko* cells. Re-probing for Tubulin (TUB) controlled loading of extracts. The Tubulin blots are related to the MGA blots. Uncropped versions of the MGA blots are shown in S4 Fig. (B) Representative genome browser screenshots of a 0.5 Mb region of chromosome 19 showing reduced binding of PRC1.6 components to several promoters in L3MBTL2*ko* and in E2F6*ko* cells but not in PCGF6*ko* cells. (C) Heat map views of the distribution of MGA, L3MBTL2 and E2F6 peaks in wild type cells (n = 8342) and in MGA*ko*, L3MBTL2*ko*, E2F6*ko* and PCGF6*ko* cells at +/- 2 kb regions centred over the MGA peaks. (D) Scatter plots comparing the signal intensity of MGA, L3MBTL2 and E2F6 peaks in wild type cells with the signal intensity of corresponding peaks in L3MBTL2*ko* (left panels), E2F6*ko* (middle panels) or PCGF6*ko* cells (right panels). Normalized ChIP-seq read counts in MGA ChIP-seq peak regions of wild type cells were plotted against the normalized read counts in corresponding peak regions of L3MBTL2*ko*, E2F6*ko* or PCGF6*ko* cells. (E) Left panel, scatter plot showing the correlation between reduced MGA binding and reduced L3MBTL2 binding in E2F6*ko* cells. Right panel, scatter plot showing the correlation between reduced MGA binding and reduced E2F6 binding in L3MBTL*ko* cells. The top 500 ranked MGA binding sites were used to calculate the fold change of normalized ChIP-seq read counts in L3MBTL2*ko* and E2F6*ko* cells relative to wild type cells.

As MGA is able to bind a subset of PRC1.6 loci independent of its DNA-binding activity, we investigated the potential contribution of E2F6, L3MBTL2 or PCGF6 to the recruitment of PRC1.6 to its target sites. To address this issue, we profiled binding of MGA, L3MBTL2 and E2F6 in cells lacking L3MBTL2, E2F6 or PCGF6 (L3MBTL2*ko*, E2F6*ko* or PCGF6*ko* cells). Importantly, the level of MGA and of other PRC1.6 subunits in L3MBTL2*ko-*, E2F6*ko-*, and PCGF6*ko* cells was unaffected (Fig 4A and S4 Fig). Analysis of the ChIP-seq data sets revealed that the overall genomic positions of the PRC1.6 binding sites in E2F6*ko-*, L3MBTL2*ko-* and PCGF6*ko* cells are similar to those in wild type cells (Fig 4B and 4C). However, the signal strengths of the MGA and L3MBTL2 peaks in E2F6*ko* cells and the signal strength of the MGA and E2F6 peaks in L3MBTL2*ko* cells were significantly reduced, but only slightly affected in PCGF6*ko* cells (Fig 4C and 4D). Notably the extent of reduction of MGA binding in E2F6*ko* cells correlated well with the extent of reduction of L3MBTL2 binding in E2F6*ko* cells (Fig 4E, left panel). Equally, the extent of reduction of MGA binding in L3MBTL2*ko* cells correlated well with the extent of reduction of E2F6 binding in L3MBTL2*ko* cells (Fig 4E, right panel). These results demonstrate that the genomic localization of PCR1.6 requires the simultaneous association of MGA, L3MBTL2, E2F6 and PCGF6 in a single complex.

### L3MBTL2 and E2F6 recruit PRC1.6 differently to distinct sets of genes

**Fig 5:**
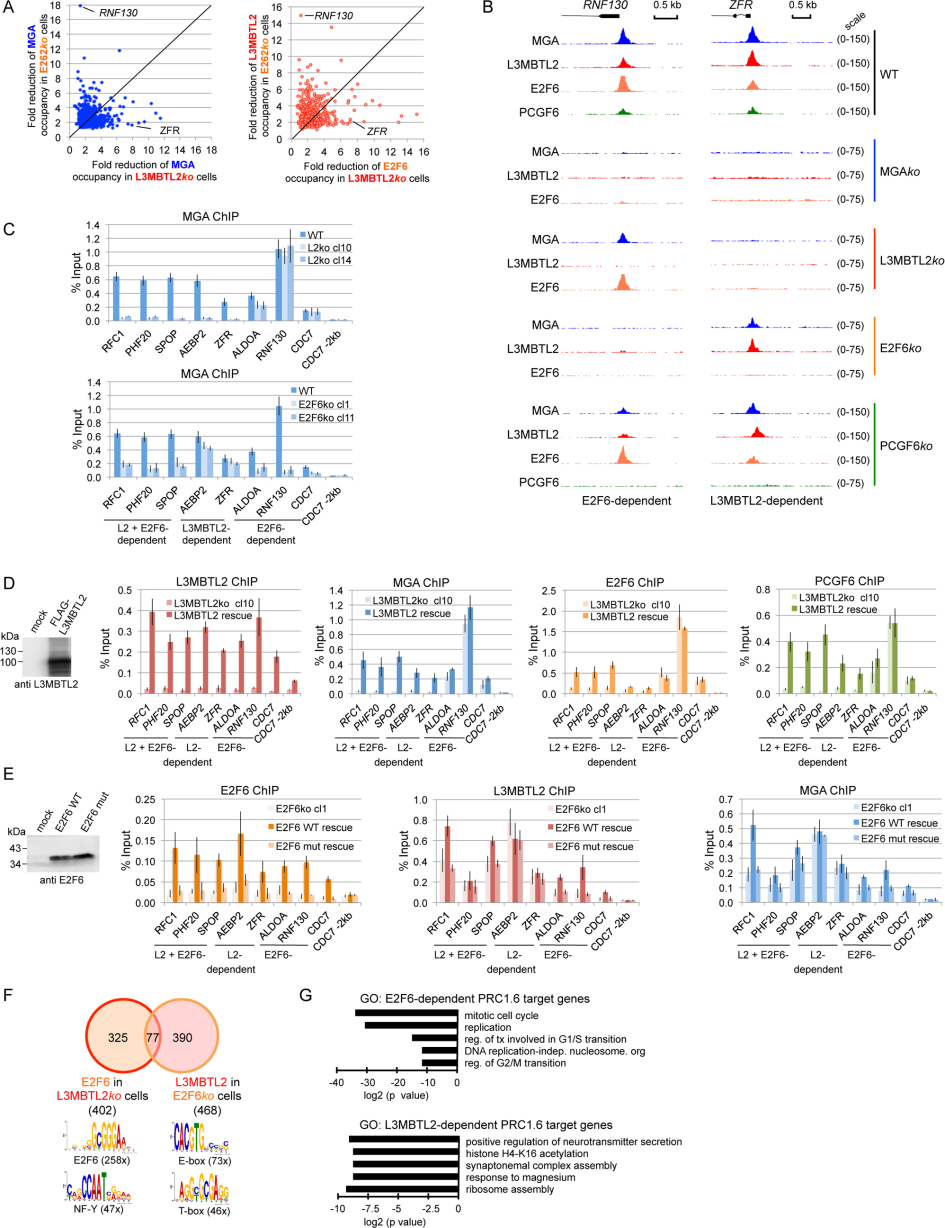
L3MBTL2 and E2F6 recruit PRC1.6 differentially in a promoter-specific manner. (A) Left panel, scatter plot comparing the extent of reduction (fold change of normalized tag counts) of MGA binding in L3MBTL2*ko* cells with the extent of reduction in E2F6*ko* cells. Right panel, scatter plot comparing the extent of reduction of E2F6 binding in L3MBTL2*ko* cells with the extent of reduction of L3MBTL2 in E2F6*ko* cells. The E2F6-dependent *RNF130* and the L3MBTL2-dependent *ZFR* promoters are indicated for clarity. (B) Genome browser screenshots of ChIP-seq tracks showing binding of MGA, L3MBTL2, E2F6 and PCGF6 to the *RNF130 and ZFR* promoters in wild type cells (WT), and in MGA*ko*, L3MBTL2*ko*, E2F6*ko* and PCGF6*ko* cells. (C) ChIP-qPCR analysis of MGA binding to selected promoters in two different L3MBTL2*ko* (L2ko cl10 and L2ko cl14, upper panel) and in two different E2F6*ko* (E2F6*ko* cl1 and E2F6*ko* cl11, lower panel) cell clones. The *CDC7* -2kb region served as a negative control region. Percent of input values represent the mean of at least three independent experiments +/- SD. (D) Expression of L3MBTL2 in L3MBTL2*ko* cells rescues binding of PRC1.6. Left, Western blot for L3MBTL2. Right, ChIP-qPCR data showing binding of exogenous L3MBTL2 and of endogenous MGA, E2F6 and PCGF6 to representative PRC1.6 target promoters. Percent of input values represent the mean of at least three independent experiments +/- SD. (E) Expression of wild type E2F6 but not of a DNA binding-deficient E2F6 mutant (E2F6mut) in E2F6*ko* cells rescues binding of PRC1.6. Left, Western blot for E2F6. Right, ChIP-qPCR data showing binding of exogenous E2F6 (wild type or DNA-binding deficient mutant) and of endogenous MGA and L3MBTL2 to representative PRC1.6 target promoters. Percent of input values represent the mean of at least three independent experiments +/- SD. (F) Venn diagram showing the overlap of E2F6 peaks in L3MBTL2*ko* cells and L3MBTL2 peaks in E2F6*ko* cells. Logos of the enriched sequence motifs were obtained by running MEME-ChIP with 300 bp summits of the ChIP-seq peaks. (G) GO analyses of biological functions of E2F6-dependent and of L3MBTL2-dependent PRC1.6 target genes. Enriched GO terms were retrieved using Enrichr. p values are plotted in -log2 scale.

Binding of MGA to the majority of its genomic sites was greatly reduced in L3MBTL2*ko* as well as in E2F6*ko* cells, indicating that both, L3MBTL2 and E2F6 contributed to genomic binding of PRC1.6. Importantly, however, the extent of reduction of MGA and E2F6 binding in L3MBTL2*ko* cells, and the extent of reduction of MGA and L3MBTL2 binding in E2F6*ko* cells did not correlate (Fig 5A). Rather, the shape of these plots revealed three distinct types of PRC1.6 binding site (i) loci where binding of MGA was reduced in both, L3MBTL2*ko* and E2F6*ko* cells (ii) loci where binding of MGA was reduced in L3MBTL2*ko* cells but not in E2F6*ko* cells; (iii) loci where binding of MGA was reduced in E2F6*ko* cells but not in L3MBTL2*ko* cells. Thus, we were able to identify L3MBTL2-dependent and E2F6-dependent PRC1.6 binding sites (Fig 5B and S5A Fig).

We also probed a panel of PRC1.6 target sites in two different L3MBTL2*ko* and E2F6*ko* cell clones by conventional ChIP-qPCR. We tested for the presence of MGA, L3MBTL2, E2F6 and PCGF6, MAX, RING2 and H2AK119ub1 (Fig 5C and S5B Fig). This analysis confirmed L3MBTL2- and E2F6-dependent binding of PRC1.6 to the *RFC1*, *PHF20* and *SPOP* promoters; L3MBTL2-dependent but E2F6-independent binding to the *AEBP2* and *ZFR* promoters; and E2F6-dependent but L3MBTL2-independent binding to the *ALDOA*, *RNF130* and *CDC7* promoters (S5B Fig). In all cases the levels of H2AK119ub1 correlated with PRC1.6 binding. Finally, we performed rescue experiments in which we found that expression of L3MBTL2 in L3MBTL2*ko* cells not only restored binding of ectopically expressed L3MBTL2 but also of endogenous MGA, E2F6 and PCGF6 to the L3MBTL2-dependent *RFC1*, *PHF20*, *SPOP*, *AEBP2* and *ZFR* promoters (Fig 5D). Of note, the enrichment levels observed in rescued L3MBTL2*ko* cells were approximately 1.5- to 3-fold lower than in wild type cells (compare Fig 5D with in Fig 1G). This is not surprising given that not all cells in the population express L3MBTL2 after transient transfection. Importantly, L3MBTL2 also re-occupied the E2F6-dependent *ALDOA*, *RNF130* and *CDC7* promoters; however, binding of MGA, E2F6 and PCGF6 to these promoters was not enhanced. This result strongly supports our model in which L3MBTL2 is only essential for recruitment of PRC1.6 to a subset of loci despite its presence at all PRC1.6 binding sites.

To gain further insight into the E2F6-dependent recruitment of PRC1.6, we examined whether the DNA-binding activity of E2F6 is necessary for PRC1.6 binding. Wild type E2F6 expressed in E2F6*ko* cells re-occupied all tested PRC1.6 target loci, and also resulted in slightly increased binding of endogenous MGA and L3MBTL2 to E2F6-dependent promoters but not to the L3MBTL2-dependent promoters (Fig 5E). The DNA binding-deficient E2F6 mutant (E2F6-L68E,V69F) did not bind to the E2F6-dependent promoters, and did not re-occupy the L3MBTL2-dependent promoters. This observation indicates that the DNA binding domain of E2F6 is not only necessary for DNA recognition but also for association with PRC1.6.

By ChIP-qPCR analysis of selected promoters we also validated that PCGF6 has a limited role in the recruitment of MGA, L3MBTL2 and E2F6 (S6 Fig). However, it is important to note that binding of RING2 in PCGF6*ko* cells was nearly reduced to levels at a negative control region. Consistently, H2AK119ub1 levels were also reduced at these promoters (S6 Fig). Thus, PCGF6, albeit not essential for binding site selection by PRC1.6, it is required to recruit RING2 to these loci. This observation is in line with a recent study that revealed recruitment of RING2 by a PCGF6-TET repressor fusion protein tethered to a Tet operator array *in vivo* [29].

We surveyed the DNA sequences of L3MBTL2- and the E2F6-dependent PRC1.6 loci and found specific enrichment of the E2F binding motif (GCGGGA) in the E2F6-dependent PRC1.6 binding sites, and specific enrichment of the E-box (CACGTG) and T-box (AGGC/TGC/TGAGG) binding motifs in the L3MBTL2-dependent PRC1.6 binding sites (Fig 5F). The strong association of E-box and T-box motifs with L3MBTL2-dependent PRC1.6 binding sites point to an important role of L3MBTL2 in facilitating or stabilizing an interaction of MGA/MAX with DNA.

Finally, we examined whether there are specific functional features shared amongst E2F6-dependent and L3MBTL2-dependent PRC1.6-bound genes. E2F6-dependent PRC1.6 target genes but not L3MBTL2-dependent PRC1.6 target genes were highly enriched in Gene Ontology (GO) terms related to cell cycle control (Fig 5G). This finding is in line with the role of E2F6 as an RB-independent transcriptional repressor during cell cycle progression [30]. GO terms associated with L3MBTL2-dependent PRC1.6 target genes included quite different biological processes such as “positive regulation of neurotransmitter secretion”, meiotic “synaptonemal complex assembly” and “ribosome assembly”. Altogether these results suggest that E2F6 and L3MBTL2 recruit PRC1.6 to distinct gene sets that regulate different biological processes.

### Role of PRC1.6 in HEK293 cell function

**Fig 6:**
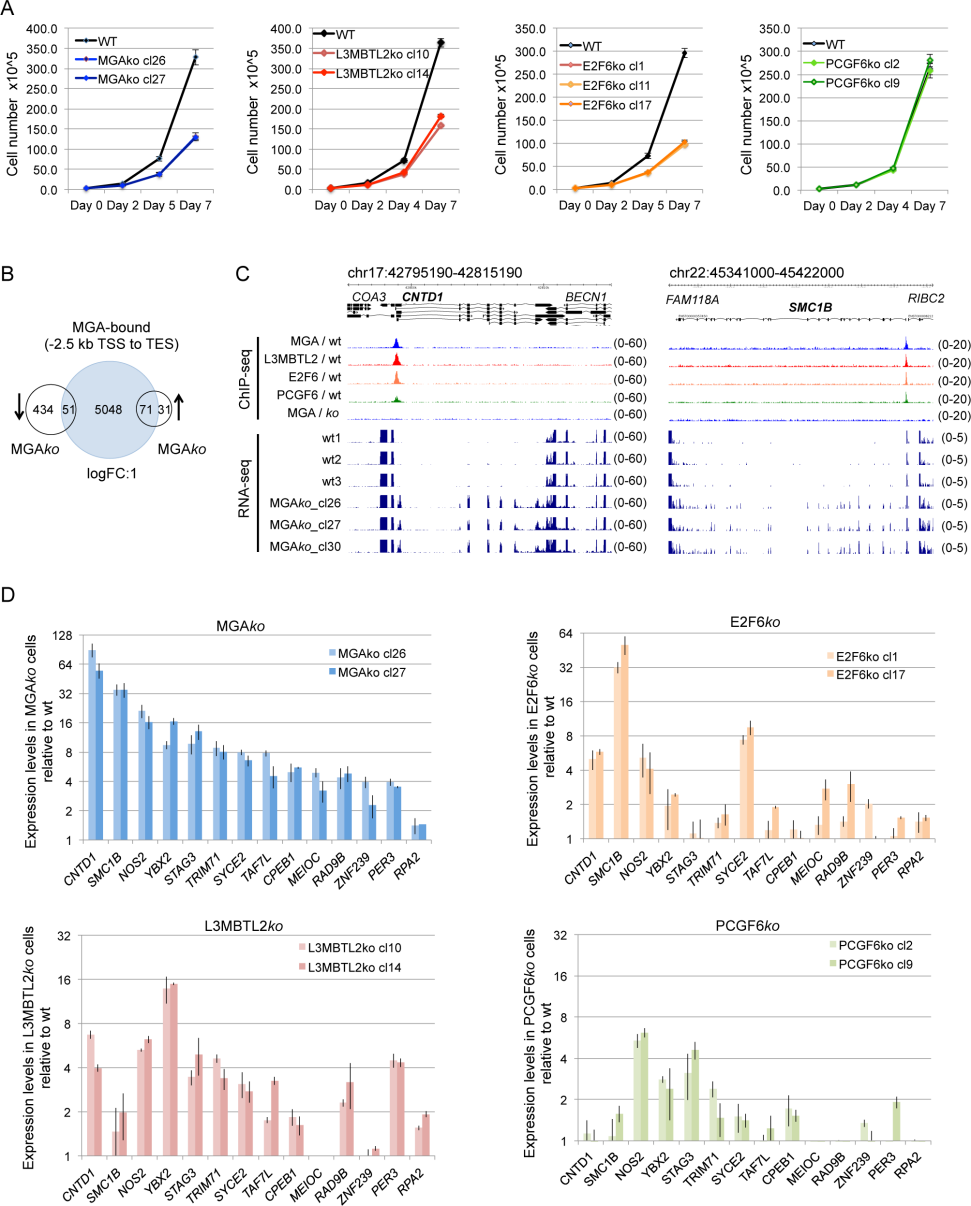
The role of PRC1.6 in HEK293 cell function. (A) Reduced proliferation of MGA*ko*, L3MBTL2*ko* and E2F6*ko* cells. Shown are growth curves of wildtype, MGA*ko*, L3MBTL2*ko*, E2F6*ko* and PCGF6*ko* HEK293 cells. Cells were seed at 3x10^5^, and counted and replated at the indicated time points. Cumulative cell numbers were calculated by multiplying the initial cell number with the fold-increase in cell numbers in each interval. (B) Venn diagrams illustrating the overlap of MGA-bound genes and genes down- or up-regulated in MGA*ko* cells. Left circle, genes with ≥2-fold reduced transcript levels in MGA*ko* cells; right circle, genes with ≥2-fold increased transcript levels in MGA*ko* cells. (C) Representative genome browser screenshots of ChIP-seq and RNA-seq tracks illustrating binding of MGA, L3MBTL2, E2F6 and PCGF6 (top tracks) to the *CNTD1* and *SMC1B* promoters, and RNA expression (bottom tracks) of the corresponding genes in three wild type samples (MGA_wt1, MGA_wt2 and MGA_wt3), and in three different MGA*ko* cell clones (MGA*ko*_cl26, MGA*ko*_cl27 and MGA*ko*_cl30). (D) RT-qPCR-based analysis of expression changes of selected genes in MGA*ko*, E2F6*ko*, L3MBTL2*ko* and PCGF6*ko* cells. Transcript levels were normalized to *B2M* transcript levels, and are depicted relative to transcript levels in wild type cells.

We went on to investigate the role of PRC1.6 in cell growth and gene expression in HEK293 cells by comparing the proliferation potential of wild type, MGA*ko*, L3MBTL2*ko*, E2F6*ko* and PCGF6*ko* cells. The growth rates of wild type cells and PCGF6*ko* cells were similar; however, we observed reduced proliferation of MGA*ko*, L3MBTL2*ko* and E2F6*ko* cells. (Fig 6A). Next, we examined the transcriptional impact of PRC1.6. RNA-seq of three independent wild type cell cultures and three independent MGA*ko* clones identified 587 genes with ≥2-fold altered expression levels in MGA*ko* cells. Expression of 485 genes was reduced in the MGA*ko* cells, while expression of 102 genes was increased (Fig 6B). Comparison of the set of de-regulated genes with the gene set bound by MGA revealed that MGA was not bound to the majority of the down-regulated genes (434/485, 89%) suggesting an indirect role of MGA in the regulation of these genes. In contrast, MGA was bound to the majority of the up-regulated genes (71/102, 70%) suggesting that PRC1.6 acts as a direct repressor on these genes. Representative ChIP-seq and RNA-seq genome browser screenshots of de-repressed genes bound by PRC1.6 are shown in Fig 6C. Interestingly, the top up-regulated genes in MGA*ko* cells included several critical regulators and effectors of meiosis such as *CNTD1*, *SMC1B*, *SYCE2*, *YBX2*, *MEIOC* (*C17orf104*), *RAD9B*, *TAF7L*, *STAG3*, *CPEB1* and *ALDH1A2*, as well as several testis-enriched genes such as *PRSS50*, *TRIM71*, *C19orf57*, *ZCWPW1*, *ZNF239*, *RIBC2*, *NEUROG2* (http://www.proteinatlas.org/).

To test whether L3MBTL2, E2F6 and PCGF6 contribute to PRC1.6 target gene repression, we examined the expression of a panel of fourteen genes in L3MBTL2*ko*, E2F6*ko* and PCGF6*ko* cells by locus-specific RT-PCR assays. We found increased transcript levels of these genes also in L3MBTL2*ko*, E2F6*ko* or PCGF6*ko* cells, yet to different degrees (Fig 6D). For example, compared with wild type cells, transcript levels of *CNTD1* were also increased in L3MBTL2*ko* and in E2F6*ko* but not in PCGF6*ko* cells. Transcript levels of *SMC1B*, however, were increased in E2F6*ko* cells but not in L3MBTL2*ko* and PCGF6*ko* cells. Conversely, transcript levels of *STAG3* were increased in L3MBTL2*ko* and PCGF6*ko* cells but not in E2F6*ko* cells (Fig 6D). Thus, L3MBTL2, E2F6 and PCGF6 contributed to repression of these genes differentially in a gene-specific manner. Interestingly, specific de-repression of these genes in L3MBTL2*ko* or E2F6*ko* cells, respectively, correlated well with the contribution of L3MBTL2 and E2F6 to PRC1.6 binding. Binding of PRC1.6 to the *CNTD1* promoter was diminished in L3MBTL2*ko* as well as in E2F6*ko* cells. Binding of PRC1.6 to the *SMC1B* promoter was lost in E2F6*ko* cells but remained in L3MBTL2*ko* cells, and binding of PRC1.6 to the *STAG3* promoter was lost in L3MBTL2*ko* cells but remained in E2F6*ko* cells (S7 Fig).

### Mga, L3mbtl2 and Pcgf6 colocalize in mouse ESCs and repress genes involved in differentiation

**Fig 7:**
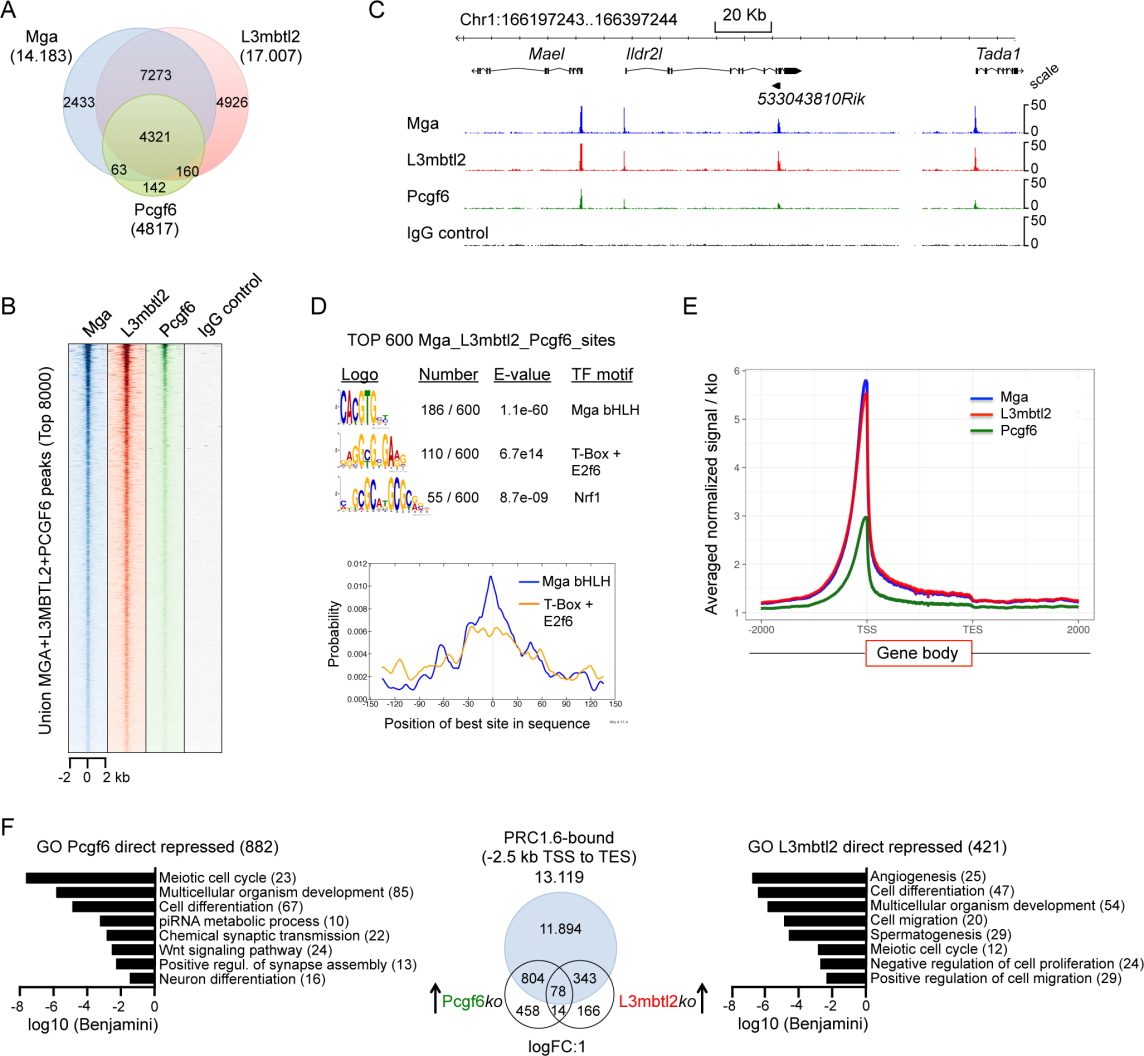
Mga, L3mbtl2 and Pcgf6 colocalize in mouse ESCs and repress genes involved in differentiation. (A) Venn diagrams representing the overlap of Mga, L3mbtl2 and Pcgf6 peaks in mouse ESCs. The total number of filtered (≥30 tags and ≥3-fold enrichment over IgG control) ChIP-seq peaks and their overlap is shown. (B) A heat map view of the distribution of the top 8000 union Mga-L3mbtl2-Pcgf6 peaks in mouse ES cells at +/- 2 kb regions centred over the MGA peaks. (C) Representative genome browser screenshot of a 100 kb region of chromosome 1 showing co-binding of Mga, L3mbtl2 and Pcgf6 to four promoter regions. (D) Sequence motifs enriched in Mga-L3mbtl2-Pcgf6 binding regions in mouse ESCs. Top, logos were obtained by running MEME-ChIP with 300 bp summits of the top 600 union Mga-L3mbtl2-Pcgf6 ChIP-seq peaks. The numbers next to the logos indicate the occurrence of the motifs, the statistical significance (E-value) and the transcription factors that bind to the motif. Bottom, local motif enrichment analysis (CentriMo) showing central enrichment of the Mga/Max bHLH domain E-box binding motif and the motif that identified MEME Tomtom as a T-box as well as a E2f6 recognition sequence. The Nrf1 motif was not centrally enriched within the 300 bp peak regions. (E) Distribution of Mga, L3mbtl2 and Pcgf6 peaks relative to positions -2000 bp upstream to +2000 bp downstream of gene bodies. TSS, transcription start site; TES, transcription end site. (F) Middle panel, Venn diagram illustrating the overlap of PRC1.6-bound genes and genes up-regulated in Pcgf6*ko* cells [26] and in L3mbtl2*ko* cells [13]. Left panel, GO analyses of biological functions of PRC1.6-bound genes that were de-repressed ≥2-fold in Pcgf6*ko* cells. Right panel, GO analyses of biological functions of PRC1.6-bound genes that were de-repressed ≥2-fold in L3mbtl2*ko* cells. Enriched GO terms were retrieved using DAVID 6.8. (GOTERM_BP_DIRECT, Functional Annotation Chart). Benjamini values are plotted in log10 scale.

Several recent studies revealed critical roles for Mga, L3mbtl2 and Pcgf6 in the regulation of mouse ES cell pluripotency, proliferation and differentiation [13,23,24,26,29]. Therefore, we investigated the genomic localization of PRC1.6 components also in mouse ES cells. We focused on Mga, L3mbtl2 and Pcgf6 as there is no available antibody, which efficiently recognizes murine E2f6. Also of note is that we failed to generate Mga-deficient ESC clones, which is in line with a previous study suggesting that Mga plays an essential role in ESCs [23]. Therefore, we used an IgG control ChIP-seq dataset as a reference for peak selection. Similar to the ChIP-seq results with chromatin of HEK293 cells, we obtained different numbers of filtered (≥30 tags and ≥3-fold enrichment over IgG) peaks for Mga (14.183), L3mbtl2 (17.007) and Pcgf6 (4817) (Fig 7A). The vast majority of the Pcgf6 peaks (90%) overlapped with Mga and L3mbtl2 peaks; and the majority of the Mga peaks (82%) overlapped with the L3mbtl2 peaks. Genome browser track and heatmap views of binding densities also revealed clear colocalization of Mga, L3mbtl2 and Pcgf6 (Fig 7B and 7C). Moreover, visual inspection of genome browser tracks did not confirm any Mga-, L3mbtl2- or Pcgf6-specific binding site (S8A Fig). Collectively, our ChIP-seq data sets reveal that Mga, L3mbtl2 and Pcgf6 colocalize in mouse ESCs suggesting strongly that the function of PRC1.6 is conserved in murine and human cells. This conclusion was further supported by a *de novo* sequence motif analysis of the top 600 ranked Mga/L3mbtl2/Pcgf6 peak regions, which revealed the presence of centrally enriched E-box as well as T-box and E2F6/DP1 recognition sequences as prevalent motifs (Fig 7D). Finally, as in HEK293 cells the majority of the Mga/L3mbtl2/Pcgf6 binding sites were located close to transcriptional start sites (Fig 7E). However we observed that the genomic distribution of the Mga/L3mbtl2/Pcgf6 peaks in mouse ESCs differ to some extent from the distribution in HEK293 cells. In many instances, we observed multiple Mga/L3mbtl2/Pcgf6 peaks within a gene locus including the promoter, exons, and the 3´-end (S8B Fig). Potentially, the peaks within gene bodies were not direct PRC1.6 binding sites but reflect local intragenic loops within genes that fold exons close to cognate promoters. The capture of such structural features by ChIP-seq has been reported previously [31]. It is also possible that these intragenic peaks reflect discrete compacted chromatin structures similar to those generated by canonical cPRC1 [32].

To examine the impact of the Mga/L3mbtl2/Pcgf6 binding sites on gene expression in mouse ESCs, we compared our ChIP-seq data sets with genes that were deregulated in L3mbtl2-depleted [13] or in Pcgf6-depleted ESCs [26]. We found that two-third of the genes (882 out of 1354) that were up-regulated in Pcgf6-depleted ES cells, and 71% of the genes that were up-regulated in L3mbtl2-depleted cells (421 out of 587) by ≥2-fold were bound by Mga, L3mbtl2 and Pcgf6 (Fig 7F). Interestingly, genes aberrantly expressed in Pcgf6*ko* and in L3mbtl2*ko* ESCs largely do not overlap (Fig 7F). Nevertheless, a GO analysis revealed that Pcgf6-dependent, as well as L3mbtl2-dependent repressed PRC1.6 target genes were strongly associated with germ-line development (meiosis and spermatogenesis). Also the small group of 78 direct PRC1.6 target genes that were up-regulated in Pcgf6*ko* as well as in L3mbtl2*ko* ES cells (Fig 7F) encode several meiotic genes including *Syce3*, *Stk31*, *Slc22a*, *Mei1* and *Tdrkh*. Remarkably, promoters of the meiotic genes were within the top ranked 200 Mga, L3mbtl2 and Pcgf6 peaks. Genes specifically de-repressed in Pcgf6-depleted ES cells but not in L3mbtl2-depleted ES cells were related to the wnt signaling pathway and to neuron differentiation. Conversely, specific L3mbtl2-repressed genes were associated with angiogenesis and positive regulation of cell migration (Fig 7F). This finding suggests that Pcgf6 and L3mbtl2 repress common as well as different sets of genes despite the presence of both factors at all target genes within the PRC1.6 complex.

### PRC1.6 distribution in mESCs partially overlaps with other PRC1 complexes

**Fig 8:**
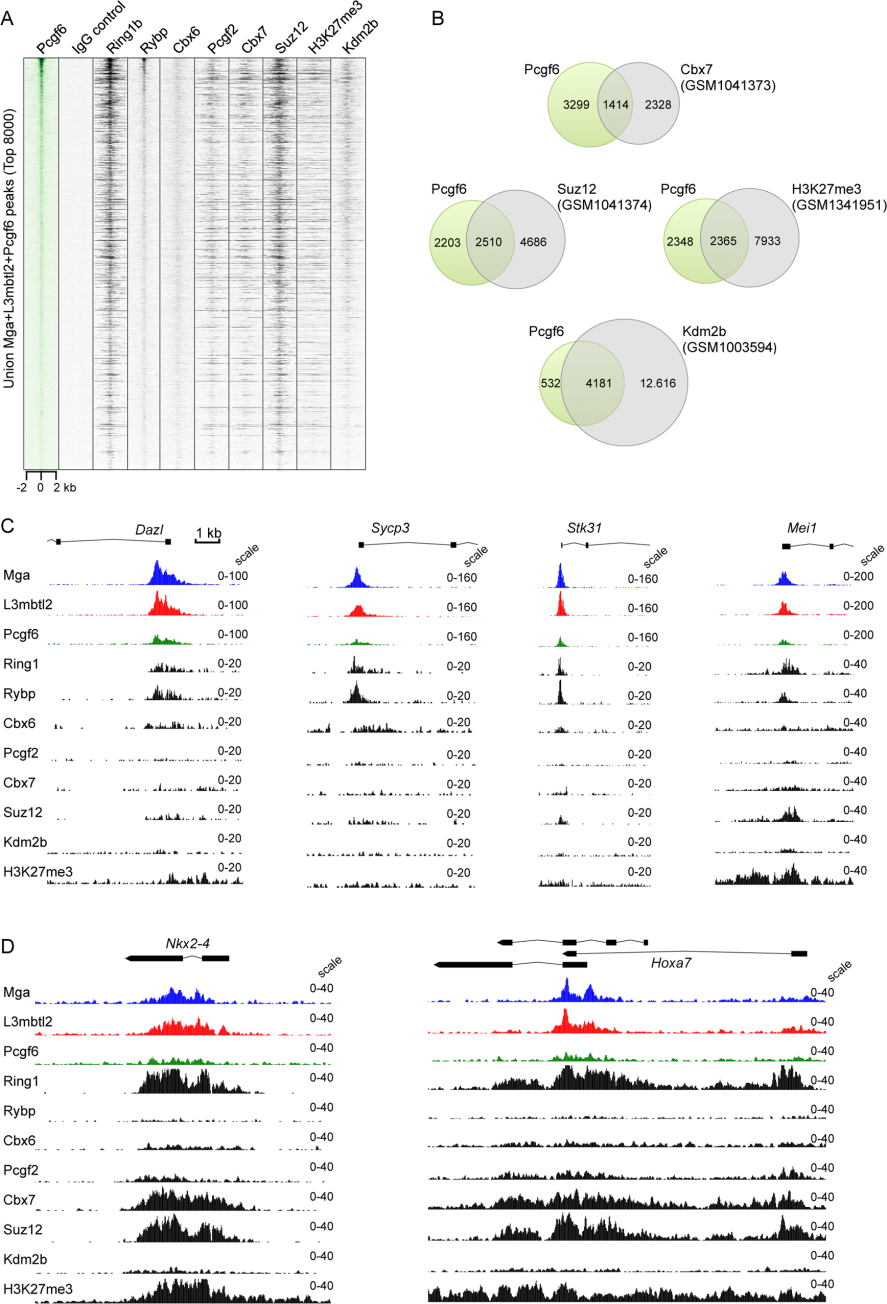
PRC1.6 binding sites partially overlap with cPRC1, PRC2 and ncPRC1.1 binding sites. (A) ChIP-seq heatmaps of Pcgf6, IgG control, Ring1b (GSM1041372) [34], Rybp (GSM1041375) [34], Cbx6-HA (GSM2610616) [33], Cbx7 (GSM2610619) [33], Pcgf2 (GSM1657387) [56], Suz12 (GSM1041374) [34], Kdm2b (GSM1003594) [6] and H3K27me3 (GSM1341951) [10] peaks in mESCs at +/- 2 kb regions centred over the Mga-L3mbtl2-Pcgf6 peaks. (B) Venn diagrams showing the overlap of high confidence Pcgf6 target genes (location of binding sites between -2.5 kb of TSS and TES) with those of Cbx7 (cPRC1), Suz12 and H3K27me3 (PRC2) and Kdm2b (ncPRC1.1). (C) Genome browser screenshots of ChIP-seq tracks at promoters of representative meiosis-related genes (*Dazl*, *Sycp3*, *Stk31 and Mei1*) (D) Genome browser screenshots of ChIP-seq tracks at cPRC1 target genes (*Nkx2-4* and *Hoxa7)*.

Mouse ESCs also express other polycomb complexes including canonical cPRC1, the non-canonical PRC1.1 (PRC1-Kdm2b, PRC1-Fbxl10) complex and PRC2. To determine whether these complexes also bind to PRC1.6 target genes we compared our Mga/L3mbtl2/Pcgf6 data sets with published ChIP-seq data sets of Ring1b, Rybp, Cbx6, Pcgf2 (Mel18), Cbx7, Suz12, H3K27me3 and Kdm2b (Fbxl10). Ring1b is a constituent of all PRC1 complexes. Cbx6 is associated with cPRC1 as well as with PRC1.6 [33]. Rybp is found in canonical as well as in non-canonical complexes; however the presence of Rybp or Cbx7 in PRC1 complexes is mutually exclusive [8,34]. Pcgf2 and Cbx7 are subunits of the cPRC1 complex and require the H3K27me3 mark to localize to chromatin [34,35]. Suz12 is a subunit of the PRC2 complex, which deposits the H3K27me3 mark. Kdm2b together with Pcgf1 forms the non-canonical variant PRC1.1 [6,9]. A heatmap view of binding densities shows tight colocalization of Rybp and Mga-L3mbtl2-Pcgf6 (Fig 8A). This is consistent with the presence of Rybp in the PRC1.6 complex [13,26]. Also the Cbx6 ChIP-seq data set displayed colocalization with Mga-L3mbtl2-Pcgf6 binding sites despite its weak density. This finding is consistent with a recent report that revealed an interaction of Cbx6 with Pcgf6 and L3mbtl2 [33]. A considerable number of PRC1.6 target regions was also occupied by canonical PRC1 (Pcgf2 and Cbx7), PRC1.1 (Kdm2b) and PRC2 (Suz12), and was decorated with H3K27me3 (Fig 8A). Unlike Rybp, however, Pcgf2, Cbx7, Kdm2b, Suz12 and H3K27me3 displayed a broader distribution at the Pcgf6 target regions. We assessed that approximately 30%, 50% and 90% of the high confidence Pcgf6 target genes were co-occupied by Cbx7 (cPRC1), Suz12/H3K27me3 (PRC2) and Kdm2b (PRC1.1), respectively (Fig 8B). Interestingly, meiosis-related genes that were de-repressed in Pcgf6- and L3mbtl2-depleted cells were largely bound exclusively by PRC1.6, whereas “typical” cPRC1 target genes such as *Nkx-2* or *Hoxa7* were decorated with both PRC1.6 and cPRC1 as well as with PRC2 (Fig 8C and 8D). Together, these results suggest that PRC1.6 and cPRC1 have both unique and common target genes in mouse ESCs.

## Discussion

**Fig 9:**
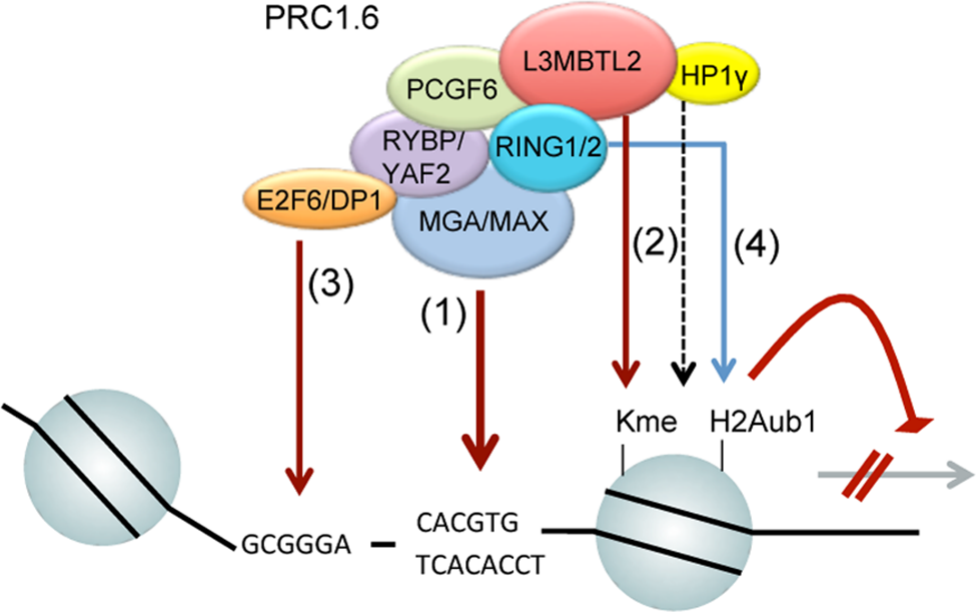
Model summarizing PRC1.6 targeting mechanisms. (1) PRC1.6 is recruited to a subset of target promoters by direct DNA binding of MGA/MAX to E-boxes (CACGTG) and/or T-boxes (TCACACCT). (2) Interaction of L3MBTL2 and HP1γ with methylated histones may promote binding site selection by facilitating and stabilizing binding of MGA/MAX. (3) MGA also acts as a scaffold tethering E2F6 that in turn mediates PRC1.6 binding to E2F6-recognition sites. (4) PCGF6 recruits RING1/2 that deposits the repressive histone mark H2AK119ub1.

In this study, we provide insights into the genomic targeting mechanism of the non-canonical PRC1 complex PRC1.6. We find that MGA, L3MBTL2, E2F6 and PCGF6 colocalize genome-wide in the context of PRC1.6. MGA is absolutely crucial for binding of the complete PRC1.6 since genome-wide binding of E2F6, L3MBTL2 and PCGF6 is lost in MGA-depleted cells (Fig 2). Mechanistically, we provide strong evidence that MGA executes recruitment of PRC1.6 to its target sites through two distinct functions (Fig 9). On the one hand, MGA acts as a sequence-specific DNA-binding factor mediating recruitment of PRC1.6 to E-box and T-box containing promoters. On the other hand, MGA has a scaffolding function, which is independent of its DNA binding capacity (Fig 3B and 3C). The scaffolding function of MGA may protect E2F6 and PCGF6 against degradation (Fig 2C).

The other components of PRC1.6 have distinct functional roles. L3MBTL2 is also involved in genomic targeting of PRC1.6 since in L3MBTL2*ko* cells, MGA, E2F6 and PCGF6 fail to bind to a large fraction of promoters (Figs 4 and 5). These L3MBTL2-dependent PRC1.6 binding sites are enriched for the bHLH E-box motif but not for the E2F6-binding motif (Fig 5). The MBT domains of L3MBTL2 are known to bind preferentially mono-, and di-methylated histone H3 and H4 marks [19–21]; and full-length L3MBTL2 interacts with histone tails independent of their lysine methylation state [12,20]. Thus, we propose that L3MBTL2 promotes binding site selection of PRC1.6 by facilitating and stabilising the interaction of MGA/MAX with E- or T-box-containing promoters.

PCGF6 has a minor role in genomic loading of PRC1.6 (Fig 4), but significantly it interacts with RING1B [36], recruits it to genomic PRC1.6 sites and facilitates downstream H2AK119 ubiquitination and transcriptional repression [29]. Consistently, PRC1.6-bound promoters are enriched of H2AK119ub1 (Fig 2E and S6 Fig). Importantly, these targets are not bound by PRC2 and are not enriched in H3K27me3 (Fig 2F). The absence of PRC2 and H3K27me3 at PRC1.6 target promoters is consistent with a previous report that revealed lack of H3K27me3 at L3MBTL2-E2F6 binding sites in K562 cells [12]. Yet, H3K27me3 enrichment was found at a subgroup of Pcgf6 binding sites in mouse ESCs (Fig 8 and [29]) suggesting a potential cell type-specific role of Pcgf6-dependent recruitment of PRC2 and downstream H3K27me3 deposition.

A large fraction of PRC1.6 binds to promoters that regulate mitotic cell cycle genes. Since binding is largely unaffected in L3MBTL2-depleted cells targeting of PRC1.6 to this class of genes is more likely mediated by E2F6 (Fig 5F and 5G). This finding is consistent with a previous report showing that E2F6 functions as an RB-independent transcriptional repressor by controlling E2F1-3-dependent transcription during cell cycle progression, particularly by counteracting the activating E2Fs during S phase [30]. These cell cycle-regulated genes are not upregulated in MGA-depleted cells. Likely, E2F4, another repressive E2F family member, compensates for the loss of E2F6-mediated PRC1.6 binding. Indeed, it has been shown that only simultaneous inhibition of both, E2F6 and E2F4 activity, results in depression of these PRC1.6 target genes [30].

Mga, L3mbtl2 and Pcgf6 colocalize also in mouse ESCs (Fig 7) strongly suggesting that the core components of PRC1.6 are evolutionarily and functionally conserved. In addition, knockdown of Mga and Max in mouse ESCs leads to the loss of Pcgf6 binding at several promoters of the genes that are up-regulated in Pcgf6*ko* cells [29] indicating that also the recruitment mechanisms in ESCs are similar to those observed in human cells. Notably, *Pcgf6* is the most highly expressed *Pcgf* paralog in undifferentiated ESCs [24], and Pcgf6 is the predominant Ring1b-interactor in ESCs [37]. These observations indicate that PRC1.6 is a major PRC1 complex in ESCs. PRC1.6 components play essential roles in ESCs including regulation of ESC pluripotency, proliferation and differentiation. Most significantly, Mga depletion leads to the death of proliferating pluripotent ICM cells *in vivo* and *in vitro*, and the death of ESCs *in vitro* [23]. Also Pcgf6*ko* and L3mbtl2*ko* as well as Max*ko* ESCs have defects in proliferation and differentiation [13,26,38] but less severe as Mga*ko* ESCs. The most severe phenotype of Mga*ko* ESCs is in line with the crucial importance of Mga for genomic PRC1.6 binding.

Consistent with published reports, we have found that in ESCs, PRC1.6 is involved in the repression of meiotic genes. Ablation of Max, the dimerization partner of Mga, activates meiotic genes in ESCs and induces cytological changes, which are reminiscent of germ cells at the leptotene and zygotene stages of meiosis [39,40]. Meiotic and germ-line-specific genes are also activated in Pcgf6*ko* and L3mbtl2*ko* cells [13,26]. Mga, L3mbtl2 and Pcgf6 bind to the promoters of these meiosis-specific genes (Fig 7) strongly suggesting that PRC1.6 directly represses these genes in ESCs thereby safeguarding/preventing meiosis. Interestingly, several meiotic genes are also de-repressed in MGA-deficient 293 cells (Fig 6). De-repression of a limited number of meiotic and germ cell-specific genes is also observed in E2F6-deficient MEFs indicating that the repressive function operates in somatic cells [41–43].

Knockdown of Pcgf6 results also in strongly increased expression levels of several mesodermal genes including *T* (Brachyury), the *Runx* transcription factor *Mlf1* and the vascular endothelial growth factor receptor 2 (Vegfr-2, Flk) encoded by the *Kdr* gene [24]. Our ChIP-seq data revealed binding of Mga, L3mbtl2 and Pcgf6 to these genes suggesting that PRC1.6 also directly represses mesodermal lineage genes in mouse ESCs.

Apart from meiosis-specific and germ-line-specific genes, quite different gene sets are de-repressed on Pcgf6- and L3mbtl2-depletion in ESCs. Based on this observation it was suggested that Pcgf6 acts independently of L3mbtl2 [24]. However we provide strong evidence that all Pcgf6 binding sites are also bound by L3mbtl2. We speculate that L3mbtl2 facilitates binding of PRC1.6 to specific loci, while Pcgf6 acts through recruitment of Ring1b and downstream H2AK119ub1 [29]. Since L3mbtl2 associates with the methyltransferases G9A and GLP [13,14] it may also facilitate H3K9 dimethylation. Indeed, G9A and GLP are required for repression of germ cell-specific genes [44]. It is also possible that L3mbtl2, known to compact nucleosomal arrays *in vitro* [12], represses transcription directly by chromatin compaction making promoters inaccessible for the transcription machinery.

## Materials and methods

### Antibodies

Rabbit polyclonal antibodies against MGA for use in ChIP experiments and immunoblotting were generated by immunizing with a bacterially expressed GST fusion protein carrying the 300 C-terminal amino acids of human MGA. Immunization was carried out by Eurogentec (Seraing, Belgium) using the 28-day Speedy immunization protocol. Antisera were affinity-purified according to a protocol described in [45] using the matrix-coupled GST-MGA fusion protein. The commercially available antibodies used in this study are shown in Table 1.

**Table 1:** Commercially available antibodies used in this study.

Antibody	Company	Cat-No.	Amount used for ChIP	WB dilution
anti-EZH2	Diagenode (Seraing, Belgium)	C15410039	3 μg	-
anti-E2F6	Santa Cruz (Dallas, TX)	sc-22823	3 μg	1:1000
anti-H2AK119Ub1	Cell Signaling (Danvers, MA)	8240	6 μL	1:2000
anti-H2B	Merck Millipore (Billerica, MA)	07–371	-	1:2000
anti-H3K27me3	Diagenode (Seraing, Belgium)	C15410195	2 μg	-
anti-HP1γ	Merck Millipore (Billerica, MA)	05–690	3 μg	-
anti-L3MBTL2	Active Motif (Carlsbad, CA)	39569	8 μL	1:2000
anti-MAX	Santa Cruz (Dallas, TX)	sc-197	3 μg	-
anti-PCGF6	Proteintech (Rosemont, IL)	24103-1-AP	3 μg	1:1000
anti-RING2	Abcam (Cambridge, UK)	ab101273	3 μg	1:2000
anti-RYBP	Sigma Aldrich (St. Louis, MO)	PRS2227	3 μg	-
anti-Tubulin	Merck Millipore (Billerica, MA)	MAB3408	-	1:5000
Rabbit IgG control	Diagenode (Seraing, Belgium)	C15410206	3 μg	-

### Generation of MGA-, E2F6-, L3MBTL2- and PCGF6-depleted cell lines

HEK293 cells were transfected using FugeneHD (Promega, Madison, WI) with plasmids expressing mammalian-codon optimized Cas9 and sgRNAs targeting the coding region of human MGA, E2F6, L3MBTL2 or PCGF6 (S1 Fig). The parental vector pSpCas9-2A-Puro (pX459) was a gift from Feng Zhang (Addgene plasmid # 48139) [46]. The sequences of the oligonucleotides used for targeting *MGA*, *L3MBTL2*, *E2F6* and *PCGF6* were as follows. *MGA*-gRNA6: CATCTGGAAAGGTACTCCCA, *MGA*-gRNA7: GTCATACTTGAATTGTATAC; *L3MBTL2*-gRNA6: GGATGTGATGAAAGGGATGA, *L3MBTL2-*gRNA7: GCCTCTGTCATCCAGACAGC; *E2F6-*gRNA1: GGGTATTCTTGACTTAAACA, *E2F6-*gRNA2: GTTTAAGTCAAGAATACCCC, *E2F6-*gRNA3: GTCGATTCCATCTAAGACAT; *PCGF6*-gRNA3: GGTATGAAGACATTCTGTGA, *PCGF6*-gRNA4: TGTACTACTATATTGCATTT.

The empty pX459 vector was transfected as a negative control. Puromycin selection (3 μg/ml) was carried out 48 hours after transfection for 3 to 6 days. Individual colonies were isolated and the targeted loci were genotyped by PCR (see S1 Fig) and sequenced. Cell clones with indels in the targeted locus were further analyzed by Western blotting.

### Construction of expression vectors

The expression vector for 3xFLAG-L3MBTL2 has been described in [20]; and the expression vectors for HA-tagged wild type E2F6 and the DNA-binding-deficient E2F6 mutant in [18]. The HA-tag was removed by BamHI/HindIII digestion and blunt-end re-ligation. For expression of 3xFLAG-tagged MGA under control of the CMV promoter, several MGA cDNA fragments were amplified from poly(A)- and random-primed HEK293 cell cDNA libraries, and placed stepwise into pN3-3xFLAG using conventional restriction cloning procedures. The sequence of the cloned *MGA* cDNA is identical to the NCBI reference sequence XM_005254246.2 and encodes the 3115 amino acid full-length MGA isoform XP_005254303.1. Mutations of the MGA T-box and bHLH domains were introduced into the wild type *MGA* construct by replacing appropriate wild type fragments with corresponding mutant gBlock DNA fragments (IDT, Leuven, Belgium) using internal restriction sites of the *MGA* cDNA.

### Expression of MGA, L3MBTL2 and E2F6

For expression of MGA, L3MBTL2 or E2F6, the respective knockout clones were transiently transfected with the corresponding expression plasmid using the FugeneHD transfection reagent (Promega). Five million cells on a 15-cm dish were transfected with 20 μg of plasmid DNA, harvested 48 hours after transfection and cross-linked chromatin was prepared. Expression of the proteins was monitored by Western blotting.

### Cell growth conditions and growth curves

HEK293 cells were cultured in DMEM/F-12 + GlutaMax medium (Gibco, Thermo Fisher, Waltham, MA) supplemented with 10% fetal bovine serum (Sigma Aldrich, St. Louis, MO) and 1% Penicillin/Streptomycin (Sigma Aldrich). Mouse J1 ES cells were cultivated feeder-cell free on gelatin-coated plates in DMEM + GlutaMax (Gibco, Thermo Fisher), supplemented with 15% fetal bovine serum (Biochrom, Berlin, Germany), 1% non-essential amino acids (Gibco, Thermo Fisher), 1% Penicillin/Streptomycin (Sigma Aldrich), 50 mM ß-Mercaptoethanol and 1000 U/mL ESGRO leukemia inhibitory factor (Merck Millipore, Billerica, MA). For determination of growth rates of wild type and corresponding knockout HEK293 cell lines, 3x10^5^ cells were plated on a 6-well dish and counted in two or three days intervals as indicated in Fig 6A. Cumulative cell numbers were calculated by multiplying the initial cell number with the fold-increase in cell numbers in each interval.

### ChIP-qPCR

ChIP experiments were performed with the One Day ChIP kit (Diagenode, Seraing, Belgium). ChIP-qPCRs with gene-specific primers (Table 2) were performed using the ImmoMix PCR reagent (Bioline, Luckenwalde, Germany) in the presence of 0.1 x SYBRGreen (Molecular Probes, Thermo Fisher, Waltham, MA). Enrichment was calculated relative to input.

**Table 2:** Primers for ChIP-qPCR analyses.

Promoter	Forward Primer 5´-3´	Reverse Primer 5´-3´
*hAEBP2*	AGGGGACACTCCTCAAACAC	CTGCAAGTGCTCAGCGTTTC
*hALDOA*	GACCAGAGCGGTGTTTGTAC	GACCAGAGCGGTGTTTGTAC
*hCCND2*	TGACGGGAGGAAGGAGGTGA	GCAAACACCACCACCCCTTC
*hCDC7*	GAGCCACAGAAGTCGTACTC	CCGAACCAGATGCTTAGTGC
*hCDC7 -2kb*	CACCTTCTTACCTCACAGAC	GGGTATAGTTCAGGGTGAAG
*hCDIP1*	CTGGCATGAGTGAAGAGGAG	AAGGAGGCGGGTAGACTTTG
*hFUT9*	CACCCTTCCTGCTCTTCCGT	GCAGGGGAGAGCAGTGAATC
*hMYT1*	TCAGAGCAGGACACAGGACT	TGCGAACTCCTAAGCCAGCT
*hPHF20*	TGAGTGGGGACTTCGTGTTC	GACCAACCGACAGAAGGACT
*hRFC1*	GCCAAAAACCGAGCTCACAC	CCATTCGCGCCAACAACTTC
*hRNF130*	CGCCTGACAGAGAAACAACC	GGGTCAGCGGAACACAAAGT
*hRPA2*	CACGCCGAACAAAGGAAGTG	CAGTTGGCTCCAAAAGCCTC
*hSPOP*	ACCCTTGGCTTGTTACGCCT	CCGCCTATCTTTCCTAGTGC
*hTFAP4*	GCCCGGACATCTGCATTTTG	GTTGGGCAGGAGTGTCTACA
*hTSH2B*	ACGCCACTTCCCATTGTCCA	TGACACCTCCGGCATAGCTA
*hZFR*	ACTACAGCTCCCAGGATGCC	AGGGCATATGGGAATCATGG

### ChIP-seq and data analysis

Three to four individual ChIPs were pooled and purified on QIAquick columns (Qiagen, Hilden, Germany). Five nanograms of precipitated DNA were used for indexed sequencing library preparation using the Microplex library preparation kit v2 (Diagenode). Libraries were purified on AMPure magnetic beads (Beckman Coulter, Brea, CA) and quantified on a Bioanalyzer (Agilent Technologies, Santa Clara, CA). Pooled libraries were sequenced on an Illumina HiSeq1500 platform (Illumina Inc., San Diego, CA), rapid-run mode, single-read 50 bp (HiSeq SR Rapid Cluster Kit v2, HiSeq Rapid SBS Kit v2–50 cycles) according to manufacturer´s instructions.

Raw ChIP-seq reads were aligned using Subread [47] version 1.4.3-p1. Reads matching multiple locations were discarded during alignment. Peaks were called with MACS [48] version1.4.0rc2 against the respective knockout control or against IgG for mouse ES cell data. Filtered peaks were required to have at least 30 tags and a sequencing depth-corrected ratio over control of 3x. Published mESC datasets (Fig 8) were retrieved from GEO and processed as above using Subread and MACS, but were not filtered. Unions and overlaps were calculated on an ‘at least 1bp overlap’ basis. For motif search and heatmaps, peaks were centred at their summits and fixed sized regions extracted. Summits were defined as the point of highest read overlap after extending the reads to 200 bp. Heatmaps show number of reads extended to 200 bp, normalized for sequencing depth. The signal distribution was truncated at the 99^th^ percentile in each sample in order to increase contrast. Regions for heatmaps were ordered by the sum of signal in the first sample depicted. ChIP-seq signal plots shown in Figs 1F and 7E are also based on reads extended to 200 bp. Genes were associated with a peak if the peak was located within -2.5 kb of TSS to TES.

### Motif analysis

*De novo* motif search including Tomtom and CentriMo was performed online with MEME-ChIP versions 4.11.3 and 4.11.4 (http://meme-suite.org/meme_4.11.4/tools/meme-chip) [49] within the MEME Suite (http://meme-suite.org) [50] using 300 bp sequences surrounding peak summits (+/- 150 bp).

### Gene ontology analysis

Gene Ontology (GO) analyses were performed using Enrichr (http://amp.pharm.mssm.edu/Enrichr/) [51,52] and the DAVID 6.8 web-based tool (https://david.ncifcrf.gov) [53,54].

### Expression analysis

For RNA-seq, total RNA was extracted from HEK293 cells stably transfected with the empty pX459 vector and three different MGA*ko* clones by using the RNeasy Mini system (Qiagen) including an on-column DNaseI digestion. RNA integrity was assessed on an Experion (Bio-Rad Laboratories, Hercules, CA). Sequencing libraries were generated using the TruSeq stranded mRNA Library Preparation Kit (Illumina Inc.). Libraries were quantified on a Bioanalyzer (Agilent Technologies) and subsequently sequenced on an Illumina HiSeq1500 platform (Illumina Inc.), rapid-run mode, single-read 50 bp (HiSeq SR Rapid Cluster Kit v2, HiSeq Rapid SBS Kit v2–50 cycles) according to manufacturer´s instructions.

Quantitative RT-qPCR was performed essentially as described in [20]. cDNA was synthesized with the Tetro reverse transcriptase (Bioline) using one to two microgram of total RNA. Quantitative PCR was performed in triplicates by using the ImmoMix PCR reagent (Bioline) with gene-specific primers (Table 3). Values were normalized to *GAPDH and/or B2M* mRNA content.

**Table 3:** Primers for RT-qPCR analyses.

Gene	Forward Primer 5´- 3´	Reverse Primer 5´- 3´
*hB2M*	AGTATGCCTGCCGTGTGAAC	GGAGCAACCTGCTCAGATAC
*hCNTD1*	AACTCCACTCCCAGTCAGCT	CCACTCCGTGAGTCAGGATT
*hCPEB1*	TGGGTCTGACTTGGTGGACA	TGACAGAGACAGGAAGGGCA
*hGAPDH*	TGCACCACCAACTGCTTAGC	GGCATGGACTGTGGTCATGA
*hMEIOC*	GCTTCCAATCGGCAAAGGCA	ACAGGGCAGTGCGTGTTTTC
*hNOS2*	CTCCCATCCTTGCATCCTCA	AAACACCAAGGTCATGCGGC
*hPER3*	AATGTCGCCGAAGAGCCCAT	GCTCCTCCTTTTGCCCATGA
*hPHF20*	CTCCAAAAAGGCCCTACCAG	TAGTCCAGCCAGCTCTCCAA
*hRAD9B*	GAGCATCTTCACCACAGTCAC	CCACTCTTTTCATTGCAGGGC
*hRPA2*	CCAGGAATGAGTGAAGCAGG	TCAGGTACCCAGTTAGATCCA
*hSMCB1*	TTCTATTCCAGAGCCGACGC	GACTCTCCGTGTCTCTTGCT
*hSYCE2*	TAAGACTCAGATGGGGGCCA	GCAGCTGTCAGCATTCACCA
*hTAF7L*	CAGCCACAAGCAGGGTCATA	CCTCATCCTCATCCTCATCC
*hTRIM71*	ACCCATCTGTCGTGAGTGCA	ACCTCCGACTGCACAACCTT
*hYBX2*	CGTAAGTCCCGCCGATTCAT	TGGGGCTGTCTCTTTGGGTT
*hZNF239*	GTGATGCTGGGCAACTACAG	GTTCAGGCTCCCCATCCACT

### Databases and data availability

The source for genome sequences and annotation was Ensembl revision 83 [55]. Our ChIP-seq and RNA-seq were deposited at ArrayExpress under accession numbers E-MTAB-6006 (ChIP-seq, HEK293), E-MTAB-6007 (ChIP-seq, mouse ES) and E-MTAB-6005 (RNA-seq, HEK293). For assessing the overlap of PRC1.6 with other polycomb complexes in mESCs, the following ChIP-seq data sets were used: Ring1b (GSM1041372), Rybp (GSM1041375), Cbx6-HA (GSM2610616), Pcgf2 (GSM1657387); Cbx7 (GSM2610619), Suz12 (GSM1041374), Kdm2b (GSM1003594) and H3K27me3 (GSM1341951).

## Supporting information

S1 Fig Schematic presentation of the CRISPR/Cas-mediated generation of MGA*ko*, L3MBTL2*ko*, E2F6*ko* and PCGF6*ko* HEK293 cells. Shown are the genomic exon/intron structures of the human *MGA*, *L3MBTL2*, *E2F6* and *PCGF6* genes. PAM sequences are highlighted in red, and sgRNA targeting sequences are highlighted in blue. The location of the PCR primers used for amplification of the targeted loci are indicated by arrowheads. Sequencing of the PCR products identified the indicated deletions, which led to frameshift mutations and/or deletion of exon/intron boundaries.
(TIF)

S2 Fig MGA, L3MBTL2, E2F6 and PCGF6 colocalize in HEK293 cells. (A) Knockout control cell lines identified false postive peaks. Genome browser screenshots showing false positive PCGF6 and L3MBTL2 peaks at the *BMF* and *PXMP2*, and at the *SMARCD2* and *TJP3* promoters, respectively. (B, C, D, E) Venn diagramms showing the overlap of filtered (F) peaks (≥30 tags and ≥3-fold enrichment over the knockout control) with unfiltered (UF) peaks called by MACS. (B) Filtered MGA peaks were compared with unfiltered L3MBTL2, E2F6 and PCGF6 peaks. (C) Filtered L3MBTL2 peaks were compared with unfiltered MGA, E2F6 and PCGF6 peaks. (D) Filtered E2F6 peaks were compared with unfiltered MGA, L3MBTL2 and PCGF6 peaks. (E) Filtered PCGF6 peaks were compared with unfiltered MGA, L3MBTL2 and E2F6 peaks. Representative genome browser screenshots of potentially MGA-, L3MBTL2-, E2F6 or PCGF6-specific peaks are presented below the Venn diagramms.
(TIF)

S3 Fig Global H2AK119ub1 levels are similar in wild type, MGA*ko*, L3MBTL2*ko*, E2F6*ko* and PCGF6*ko* cells. (A) Coomassie Blue-stained SDS gel showing acid-extracted histones [57] of wild type (WT), L3MBTL2*ko* (L2*ko)*, MGA*ko*, E2F6*ko* and PCGF6*ko* cells. The locations of the linker histone protein H1 and the core histone proteins H2A, H2B, H3 and H4 are indicated. (B) Western blot analysis of H2AK119ub1 using the acid-extracted histone preparations shown in panel (A). (C) Re-probing for H2B controlled loading of extracts.
(TIF)

S4 Fig Expression of MGA is not affected in L3MBTL2*ko*-, E2F6*ko*, and PCGF6*ko* cells. Western blot analysis of MGA with whole cell extracts from wild type (WT), MGA*ko*, L3MBTL2*ko*, E2F6*ko* and PCGF6*ko* HEK293 cells. Shown are uncropped Western blots. The blots were stripped and re-probed with anti-Tubulin.
(TIF)

S5 Fig L3MBTL2 and E2F6 promote binding of PRC1.6 differentially in a promoter-specific manner. (A) Additional genome browser screenshots of ChIP-seq tracks showing differential binding of PRC1.6 components (MGA, L3MBTL2 and E2F6) in L3MBTL2*ko* and E2F6*ko* cells. Binding of MGA to the *LACTB2* promoter was reduced in L3MBTL2*ko* and E2F6*ko* cells. Binding of MGA to the *AEBP2 and TSSK2* promoters was lost in L3MBTL2*ko* cells but remained in E2F6*ko* cells. Conversely, binding of MGA to the *TRA2B and FOXRED2* promoters was lost in E2F6*ko* cells but remained in L3MBTL2*ko* cells. (B) Local levels of L3MBTL2, E2F6, PCGF6, MAX, RING2 and H2AK119ub1 at selected PRC1.6 target promoters were determined in two different L3MBTL2*ko* (L2ko cl10 and L2ko cl14) and in two different E2F6*ko* (E2F6*ko* cl1 and E2F6*ko* cl11) cell clones by ChIP-qPCR. The *CDC7* -2kb region served as a negative control region. Percent of input values represent the mean of at least three independent experiments +/- SD.
(TIF)

S6 Fig PCGF6 is essential for RING2 recruitment. Local levels of PCGF6, MGA, L3MBTL2, E2F6, RING2 and H2AK119ub1 at selected PRC1.6 target promoters were determined in two different PCGF6*ko* cell clones (PCGF6*ko* cl2 and PCGF6*ko* cl9) by ChIP-qPCR. The *CDC7* -2kb region served as a negative control region. Percent of input values represent the mean of at least three independent experiments +/- SD.
(TIF)

S7 Fig E2F6- and L3MBTL2-dependent binding of PRC1.6 to the meiotic *CNTD1*, *SMC1B* and *STAG3* genes. Genome browser screenshots of ChIP-seq tracks showing binding of MGA, L3MBTL2, E2F6 and PCGF6 to the *CNTD1*, *STAG3* and *SMC1B* promoters in wild type cells (WT), and in MGA*ko*, L3MBTL2*ko*, E2F6*ko* and PCGF6*ko* cells.
(TIF)

S8 Fig Mga, L3mbtl2 and Pcgf6 colocalize in mouse ESCs. (A) Top, Venn diagrams showing the overlap of filtered Mga (left), L3mbtl2 (middle) and Pcgf6 (right) MACS peaks (F; ≥30 tags and 3x over IgG) with unfiltered MACS peaks (UF) of the two other PRC1.6 subunits. Bottom, representative genome browser screenshots of ChIP-seq tracks of potential Mga-, L3mbtl2- or E2f6-specific peaks indicate also binding the other PRC1.6 subunits. (B) Genome browser screenshots of ChIP-seq tracks showing multiple Mga, L3mbtl2 and Pcgf6 peaks in promoter regions and in gene bodies. Alternative transcripts according to Ensembl are shown above.
(TIF)

## References

[1] Simon JA, Kingston RE (2013) Occupying Chromatin: Polycomb Mechanisms for Getting to Genomic Targets, Stopping Transcriptional Traffic, and Staying Put. Molecular Cell 49: 808–824. doi: 10.1016/j.molcel.2013.02.013 23473600 10.1016/j.molcel.2013.02.013 PMC3628831

[2] Pasini D, Di Croce L (2016) Emerging roles for Polycomb proteins in cancer. Curr Opin Genet Dev 36: 50–58. doi: 10.1016/j.gde.2016.03.013 27151431 10.1016/j.gde.2016.03.013

[3] Luis NM, Morey L, Di Croce L, Benitah SA (2012) Polycomb in Stem Cells: PRC1 Branches Out. Cell Stem Cell 11: 16–21. doi: 10.1016/j.stem.2012.06.005 22770239 10.1016/j.stem.2012.06.005

[4] Di Croce L, Helin K (2013) Transcriptional regulation by Polycomb group proteins. Nature Structural & Molecular Biology 20: 1147–1155. 10.1038/nsmb.2669 24096405

[5] Aranda S, Mas G, Di Croce L (2015) Regulation of gene transcription by Polycomb proteins. Sci Adv 1: e1500737 doi: 10.1126/sciadv.1500737 26665172 10.1126/sciadv.1500737 PMC4672759

[6] Farcas AM, Blackledge NP, Sudbery I, Long HK, McGouran JF, et al (2012) KDM2B links the Polycomb Repressive Complex 1 (PRC1) to recognition of CpG islands. Elife 1: e00205 doi: 10.7554/eLife.00205 23256043 10.7554/eLife.00205 PMC3524939

[7] Gao Z, Zhang J, Bonasio R, Strino F, Sawai A, et al (2012) PCGF homologs, CBX proteins, and RYBP define functionally distinct PRC1 family complexes. Mol Cell 45: 344–356. doi: 10.1016/j.molcel.2012.01.002 22325352 10.1016/j.molcel.2012.01.002 PMC3293217

[8] Tavares L, Dimitrova E, Oxley D, Webster J, Poot R, et al (2012) RYBP-PRC1 Complexes Mediate H2A Ubiquitylation at Polycomb Target Sites Independently of PRC2 and H3K27me3. Cell 148: 664–678. doi: 10.1016/j.cell.2011.12.029 22325148 10.1016/j.cell.2011.12.029 PMC3281992

[9] Wu X, Johansen JV, Helin K (2013) Fbxl10/Kdm2b recruits polycomb repressive complex 1 to CpG islands and regulates H2A ubiquitylation. Mol Cell 49: 1134–1146. doi: 10.1016/j.molcel.2013.01.016 23395003 10.1016/j.molcel.2013.01.016

[10] Blackledge NP, Farcas AM, Kondo T, King HW, McGouran JF, et al (2014) Variant PRC1 Complex-Dependent H2A Ubiquitylation Drives PRC2 Recruitment and Polycomb Domain Formation. Cell 157: 1445–1459. doi: 10.1016/j.cell.2014.05.004 24856970 10.1016/j.cell.2014.05.004 PMC4048464

[11] Almeida M, Pintacuda G, Masui O, Koseki Y, Gdula M, et al (2017) PCGF3/5-PRC1 initiates Polycomb recruitment in X chromosome inactivation. Science 356: 1081–1084. doi: 10.1126/science.aal2512 28596365 10.1126/science.aal2512 PMC6522364

[12] Trojer P, Cao AR, Gao Z, Li Y, Zhang J, et al (2011) L3MBTL2 protein acts in concert with PcG protein-mediated monoubiquitination of H2A to establish a repressive chromatin structure. Mol Cell 42: 438–450. doi: 10.1016/j.molcel.2011.04.004 21596310 10.1016/j.molcel.2011.04.004 PMC3142354

[13] Qin J, Whyte WA, Anderssen E, Apostolou E, Chen HH, et al (2012) The polycomb group protein L3mbtl2 assembles an atypical PRC1-family complex that is essential in pluripotent stem cells and early development. Cell Stem Cell 11: 319–332. doi: 10.1016/j.stem.2012.06.002 22770845 10.1016/j.stem.2012.06.002 PMC3647456

[14] Ogawa H, Ishiguro K, Gaubatz S, Livingston DM, Nakatani Y (2002) A complex with chromatin modifiers that occupies E2F- and Myc-responsive genes in G0 cells. Science 296: 1132–1136. doi: 10.1126/science.1069861 12004135 10.1126/science.1069861

[15] Hauri S, Comoglio F, Seimiya M, Gerstung M, Glatter T, et al (2016) A High-Density Map for Navigating the Human Polycomb Complexome. Cell Reports 17: 583–595. doi: 10.1016/j.celrep.2016.08.096 27705803 10.1016/j.celrep.2016.08.096

[16] Jolma A, Yan J, Whitington T, Toivonen J, Nitta KR, et al (2013) DNA-Binding Specificities of Human Transcription Factors. Cell 152: 327–339. doi: 10.1016/j.cell.2012.12.009 23332764 10.1016/j.cell.2012.12.009

[17] Hurlin PJ, Steingrimsson E, Copeland NG, Jenkins NA, Eisenman RN (1999) Mga, a dual-specificity transcription factor that interacts with Max and contains a T-domain DNA-binding motif. EMBO J 18: 7019–7028. doi: 10.1093/emboj/18.24.7019 10601024 10.1093/emboj/18.24.7019 PMC1171765

[18] Gaubatz S, Wood JG, Livingston DM (1998) Unusual proliferation arrest and transcriptional control properties of a newly discovered E2F family member, E2F-6. Proceedings of the National Academy of Sciences of the United States of America 95: 9190–9195. 9689056 10.1073/pnas.95.16.9190 PMC21314

[19] Yoo JY, Choi KC, Kang H, Kim YJ, Lee J, et al (2010) Histone deacetylase 3 is selectively involved in L3MBTL2-mediated transcriptional repression. FEBS Lett 584: 2225–2230. doi: 10.1016/j.febslet.2010.03.048 20385135 10.1016/j.febslet.2010.03.048

[20] Stielow C, Stielow B, Finkernagel F, Scharfe M, Jarek M, et al (2014) SUMOylation of the polycomb group protein L3MBTL2 facilitates repression of its target genes. Nucleic Acids Res 42: 3044–3058. doi: 10.1093/nar/gkt1317 24369422 10.1093/nar/gkt1317 PMC3950706

[21] Guo Y, Nady N, Qi C, Allali-Hassani A, Zhu H, et al (2009) Methylation-state-specific recognition of histones by the MBT repeat protein L3MBTL2. Nucleic Acids Res 37: 2204–2210. doi: 10.1093/nar/gkp086 19233876 10.1093/nar/gkp086 PMC2673432

[22] Hu G, Kim J, Xu QK, Leng YM, Orkin SH, et al (2009) A genome-wide RNAi screen identifies a new transcriptional module required for self-renewal. Genes & Development 23: 837–848. 19339689 10.1101/gad.1769609 PMC2666338

[23] Washkowitz AJ, Schall C, Zhang K, Wurst W, Floss T, et al (2015) Mga is essential for the survival of pluripotent cells during peri-implantation development. Development 142: 31–40. doi: 10.1242/dev.111104 25516968 10.1242/dev.111104 PMC4299147

[24] Zdzieblo D, Li X, Lin Q, Zenke M, Illich DJ, et al (2014) Pcgf6, a polycomb group protein, regulates mesodermal lineage differentiation in murine ESCs and functions in iPS reprogramming. Stem Cells 32: 3112–3125. doi: 10.1002/stem.1826 25187489 10.1002/stem.1826

[25] Yang CS, Chang KY, Dang J, Rana TM (2016) Polycomb Group Protein Pcgf6 Acts as a Master Regulator to Maintain Embryonic Stem Cell Identity. Sci Rep 6: 26899 doi: 10.1038/srep26899 27247273 10.1038/srep26899 PMC4888081

[26] Zhao WK, Tong H, Huang YK, Yan Y, Teng HJ, et al (2017) Essential Role for Polycomb Group Protein Pcgf6 in Embryonic Stem Cell Maintenance and a Noncanonical Polycomb Repressive Complex 1 (PRC1) Integrity. Journal of Biological Chemistry 292: 2773–2784. doi: 10.1074/jbc.M116.763961 28049731 10.1074/jbc.M116.763961 PMC5314173

[27] Ferredamare AR, Prendergast GC, Ziff EB, Burley SK (1993) Recognition by Max of Its Cognate DNA through a Dimeric B/Hlh/Z Domain. Nature 363: 38–45. doi: 10.1038/363038a0 8479534 10.1038/363038a0

[28] Brownlie P, Ceska TA, Lamers M, Romier C, Stier G, et al (1997) The crystal structure of an intact human Max-DNA complex: New insights into mechanisms of transcriptional control. Structure 5: 509–520. 9115440 10.1016/s0969-2126(97)00207-4

[29] Endoh M, Endo TA, Shinga J, Hayashi K, Farcas A, et al (2017) PCGF6-PRC1 suppresses premature differentiation of mouse embryonic stem cells by regulating germ cell-related genes. Elife 6: e21064 doi: 10.7554/eLife.21064 28304275 10.7554/eLife.21064 PMC5375644

[30] Giangrande PH, Zhu WC, Schlisio S, Sun X, Mori S, et al (2004) A role for E2F6 in distinguishing G1/S- and G2/M-specific transcription. Genes & Development 18: 2941–2951. 15574595 10.1101/gad.1239304 PMC534654

[31] Mercer TR, Edwards SL, Clark MB, Neph SJ, Wang H, et al (2013) DNase I-hypersensitive exons colocalize with promoters and distal regulatory elements. Nature Genetics 45: 852–U181. doi: 10.1038/ng.2677 23793028 10.1038/ng.2677 PMC4405174

[32] Kundu S, Ji F, Sunwoo H, Jain G, Lee JT, et al (2017) Polycomb Repressive Complex 1 Generates Discrete Compacted Domains that Change during Differentiation. Molecular Cell 65: 432–446. doi: 10.1016/j.molcel.2017.01.009 28157505 10.1016/j.molcel.2017.01.009 PMC5421375

[33] Santanach A, Blanco E, Jiang H, Molloy KR, Sanso M, et al (2017) The Polycomb group protein CBX6 is an essential regulator of embryonic stem cell identity. Nature Communications 8: 1235 doi: 10.1038/s41467-017-01464-w 29089522 10.1038/s41467-017-01464-w PMC5663739

[34] Morey L, Aloia L, Cozzuto L, Benitah SA, Di Croce L (2013) RYBP and Cbx7 Define Specific Biological Functions of Polycomb Complexes in Mouse Embryonic Stem Cells. Cell Reports 3: 60–69. doi: 10.1016/j.celrep.2012.11.026 23273917 10.1016/j.celrep.2012.11.026

[35] Morey L, Pascual G, Cozzuto L, Roma G, Wutz A, et al (2012) Nonoverlapping Functions of the Polycomb Group Cbx Family of Proteins in Embryonic Stem Cells. Cell Stem Cell 10: 47–62. doi: 10.1016/j.stem.2011.12.006 22226355 10.1016/j.stem.2011.12.006

[36] Akasaka T, Takahashi N, Suzuki M, Koseki H, Bodmer R, et al (2002) MBLR, a new RING finger protein resembling mammalian Polycomb gene products, is regulated by cell cycle-dependent phosphorylation. Genes to Cells 7: 835–850. 12167161 10.1046/j.1365-2443.2002.00565.x

[37] Kloet SL, Makowski MM, Baymaz HI, van Voorthuijsen L, Karemaker ID, et al (2016) The dynamic interactome and genomic targets of Polycomb complexes during stem-cell differentiation. Nat Struct Mol Biol 23: 682–690. doi: 10.1038/nsmb.3248 27294783 10.1038/nsmb.3248 PMC4939079

[38] Hishida T, Nozaki Y, Nakachi Y, Mizuno Y, Okazaki Y, et al (2011) Indefinite Self-Renewal of ESCs through Myc/Max Transcriptional Complex-Independent Mechanisms. Cell Stem Cell 9: 37–49. doi: 10.1016/j.stem.2011.04.020 21726832 10.1016/j.stem.2011.04.020

[39] Suzuki A, Hirasaki M, Hishida T, Wu J, Okamura D, et al (2016) Loss of MAX results in meiotic entry in mouse embryonic and germline stem cells. Nat Commun 7: 11056 doi: 10.1038/ncomms11056 27025988 10.1038/ncomms11056 PMC4820925

[40] Okuda A, Suzuki A (2016) Unexpected link between MAX and meiotic onset. Cell Cycle 15: 2235–2236. doi: 10.1080/15384101.2016.1194137 27267300 10.1080/15384101.2016.1194137 PMC5004695

[41] Kehoe SM, Oka M, Hankowski KE, Reichert N, Garcia S, et al (2008) A conserved E2F6-binding element in murine meiosis-specific gene promoters. Biol Reprod 79: 921–930. doi: 10.1095/biolreprod.108.067645 18667754 10.1095/biolreprod.108.067645 PMC2715002

[42] Pohlers M, Truss M, Frede U, Scholz A, Strehle M, et al (2005) A role for E2F6 in the restriction of male-germ-cell-specific gene expression. Curr Biol 15: 1051–1057. doi: 10.1016/j.cub.2005.04.060 15936277 10.1016/j.cub.2005.04.060

[43] Storre J, Schafer A, Reichert N, Barbero JL, Hauser S, et al (2005) Silencing of the meiotic genes SMC1beta and STAG3 in somatic cells by E2F6. J Biol Chem 280: 41380–41386. doi: 10.1074/jbc.M506797200 16236716 10.1074/jbc.M506797200

[44] Maeda I, Okamura D, Tokitake Y, Ikeda M, Kawaguchi H, et al (2013) Max is a repressor of germ cell-related gene expression in mouse embryonic stem cells. Nat Commun 4: 1754 doi: 10.1038/ncomms2780 23612295 10.1038/ncomms2780

[45] Chalkley GE, Verrijzer CP (2004) Immuno-depletion and purification strategies to study chromatin-remodeling factors in vitro. Chromatin and Chromatin Remodeling Enzymes, Pt C 377: 421–442. 10.1016/S0076-6879(03)77028-1 14979043

[46] Ran FA, Hsu PD, Wright J, Agarwala V, Scott DA, et al (2013) Genome engineering using the CRISPR-Cas9 system. Nature Protocols 8: 2281–2308. doi: 10.1038/nprot.2013.143 24157548 10.1038/nprot.2013.143 PMC3969860

[47] Liao Y, Smyth GK, Shi W (2013) The Subread aligner: fast, accurate and scalable read mapping by seed-and-vote. Nucleic Acids Res 41: e108 doi: 10.1093/nar/gkt214 23558742 10.1093/nar/gkt214 PMC3664803

[48] Zhang Y, Liu T, Meyer CA, Eeckhoute J, Johnson DS, et al (2008) Model-based analysis of ChIP-Seq (MACS). Genome Biol 9: R137 doi: 10.1186/gb-2008-9-9-r137 18798982 10.1186/gb-2008-9-9-r137 PMC2592715

[49] Machanick P, Bailey TL (2011) MEME-ChIP: motif analysis of large DNA datasets. Bioinformatics 27: 1696–1697. doi: 10.1093/bioinformatics/btr189 21486936 10.1093/bioinformatics/btr189 PMC3106185

[50] Bailey TL, Boden M, Buske FA, Frith M, Grant CE, et al (2009) MEME SUITE: tools for motif discovery and searching. Nucleic Acids Res 37: W202–208. doi: 10.1093/nar/gkp335 19458158 10.1093/nar/gkp335 PMC2703892

[51] Chen EY, Tan CM, Kou Y, Duan QN, Wang ZC, et al (2013) Enrichr: interactive and collaborative HTML5 gene list enrichment analysis tool. Bmc Bioinformatics 14. 10.1186/1471-2105-14-128 PMC3637064 23586463

[52] Kuleshov MV, Jones MR, Rouillard AD, Fernandez NF, Duan QN, et al (2016) Enrichr: a comprehensive gene set enrichment analysis web server 2016 update. Nucleic Acids Research 44: W90–W97. doi: 10.1093/nar/gkw377 27141961 10.1093/nar/gkw377 PMC4987924

[53] Huang DW, Sherman BT, Lempicki RA (2009) Systematic and integrative analysis of large gene lists using DAVID bioinformatics resources. Nature Protocols 4: 44–57. doi: 10.1038/nprot.2008.211 19131956 10.1038/nprot.2008.211

[54] Huang DW, Sherman BT, Lempicki RA (2009) Bioinformatics enrichment tools: paths toward the comprehensive functional analysis of large gene lists. Nucleic Acids Research 37: 1–13. doi: 10.1093/nar/gkn923 19033363 10.1093/nar/gkn923 PMC2615629

[55] Cunningham F, Amode MR, Barrell D, Beal K, Billis K, et al (2015) Ensembl 2015. Nucleic Acids Research 43: D662–D669. doi: 10.1093/nar/gku1010 25352552 10.1093/nar/gku1010 PMC4383879

[56] Morey L, Santanach A, Blanco E, Aloia L, Nora EP, et al (2015) Polycomb Regulates Mesoderm Cell Fate-Specification in Embryonic Stem Cells through Activation and Repression Mechanisms. Cell Stem Cell 17: 300–315. doi: 10.1016/j.stem.2015.08.009 26340528 10.1016/j.stem.2015.08.009

[57] Shechter D, Dormann HL, Allis CD, Hake SB (2007) Extraction, purification and analysis of histones. Nature Protocols 2: 1445–1457. doi: 10.1038/nprot.2007.202 17545981 10.1038/nprot.2007.202

