# Supplementary figures and images for: MGA, L3MBTL2 and E2F6 determine genomic binding of the non-canonical Polycomb repressive complex PRC1.6
Source: PLoS Genet. 2018 Jan 30;14(1):e1007193. doi: 10.1371/journal.pgen.1007193 (PMC5806899; doi:10.1371/journal.pgen.1007193)

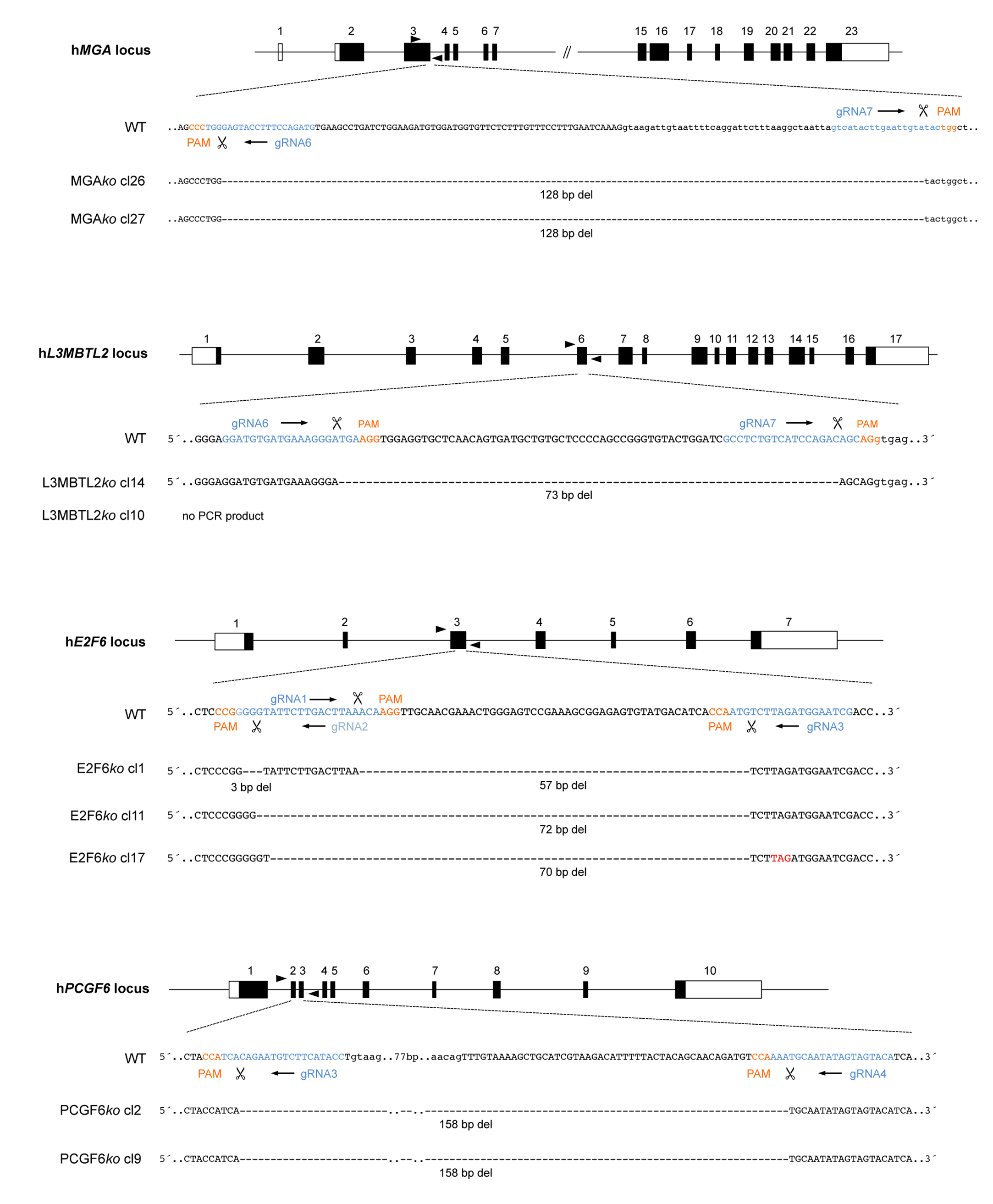

Supplement: S1 Fig — Shown are the genomic exon/intron structures of the human MGA, L3MBTL2, E2F6 and PCGF6 genes. PAM sequences are highlighted in red, and sgRNA targeting sequences are highlighted in blue. The location of the PCR primers used for amplification of the targeted loci are indicated by arrowheads. Sequencing of the PCR products identified the indicated deletions, which led to frameshift mutations and/or deletion of exon/intron boundaries. (TIF) [file pgen.1007193.s001.tif]

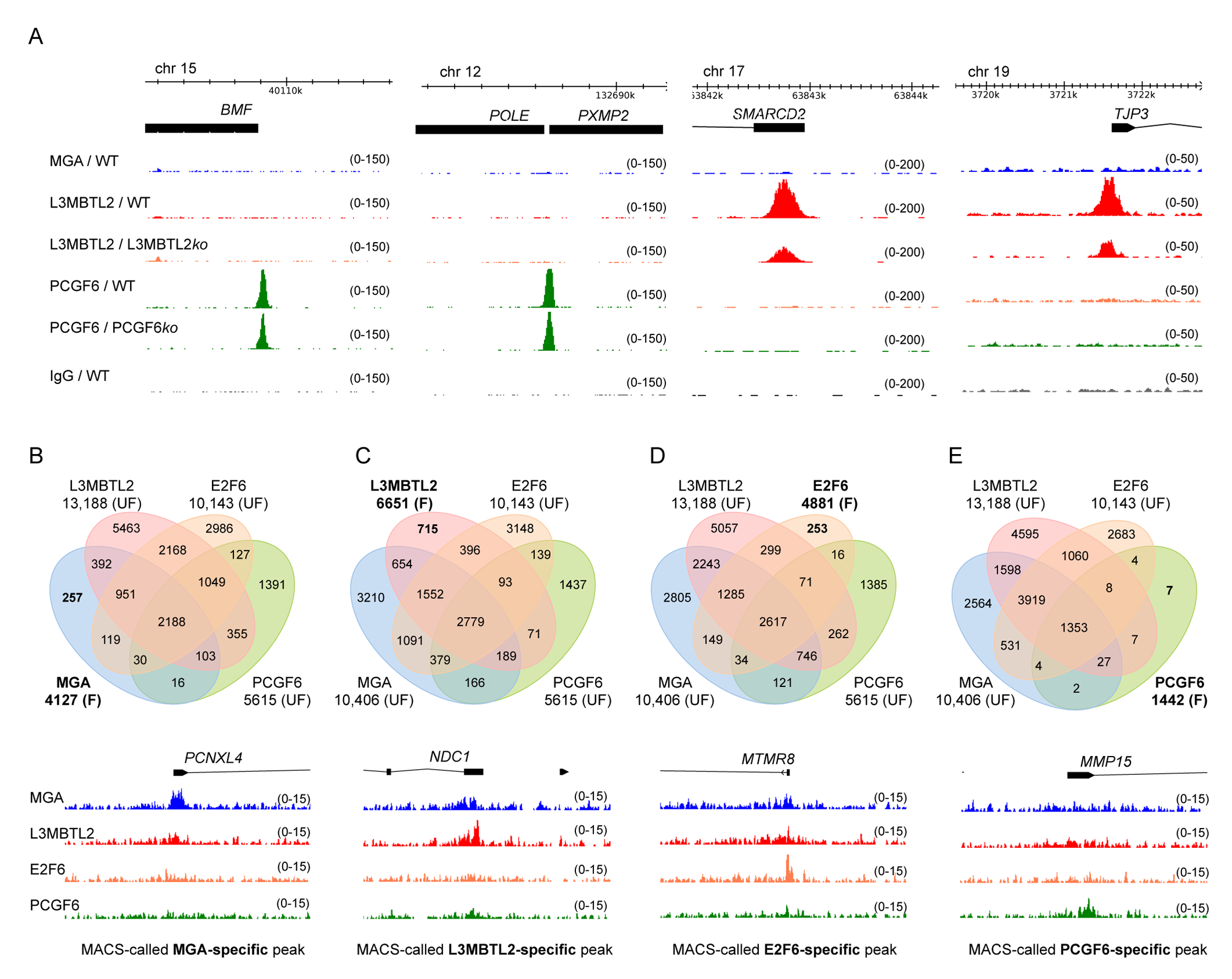

Supplement: S2 Fig — (A) Knockout control cell lines identified false postive peaks. Genome browser screenshots showing false positive PCGF6 and L3MBTL2 peaks at the BMF and PXMP2, and at the SMARCD2 and TJP3 promoters, respectively. (B, C, D, E) Venn diagramms showing the overlap of filtered (F) peaks (≥30 tags and ≥3-fold enrichment over the knockout control) with unfiltered (UF) peaks called by MACS. (B) Filtered MGA peaks were compared with unfiltered L3MBTL2, E2F6 and PCGF6 peaks. (C) Filtered L3MBTL2 peaks were compared with unfiltered MGA, E2F6 and PCGF6 peaks. (D) Filtered E2F6 peaks were compared with unfiltered MGA, L3MBTL2 and PCGF6 peaks. (E) Filtered PCGF6 peaks were compared with unfiltered MGA, L3MBTL2 and E2F6 peaks. Representative genome browser screenshots of potentially MGA-, L3MBTL2-, E2F6 or PCGF6-specific peaks are presented below the Venn diagramms. (TIF) [file pgen.1007193.s002.tif]

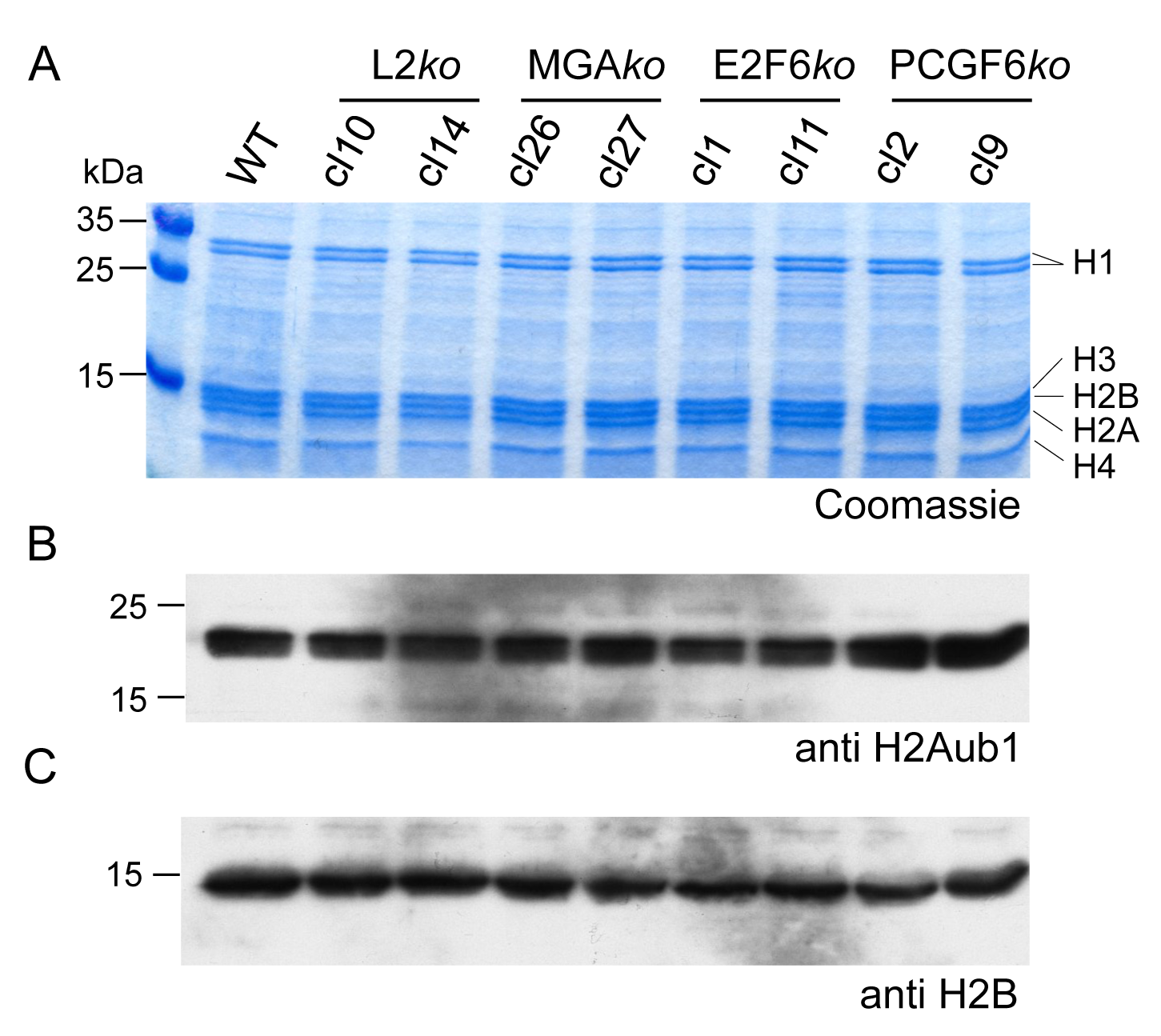

Supplement: S3 Fig — (A) Coomassie Blue-stained SDS gel showing acid-extracted histones [57] of wild type (WT), L3MBTL2ko (L2ko), MGAko, E2F6ko and PCGF6ko cells. The locations of the linker histone protein H1 and the core histone proteins H2A, H2B, H3 and H4 are indicated. (B) Western blot analysis of H2AK119ub1 using the acid-extracted histone preparations shown in panel (A). (C) Re-probing for H2B controlled loading of extracts. (TIF) [file pgen.1007193.s003.tif]

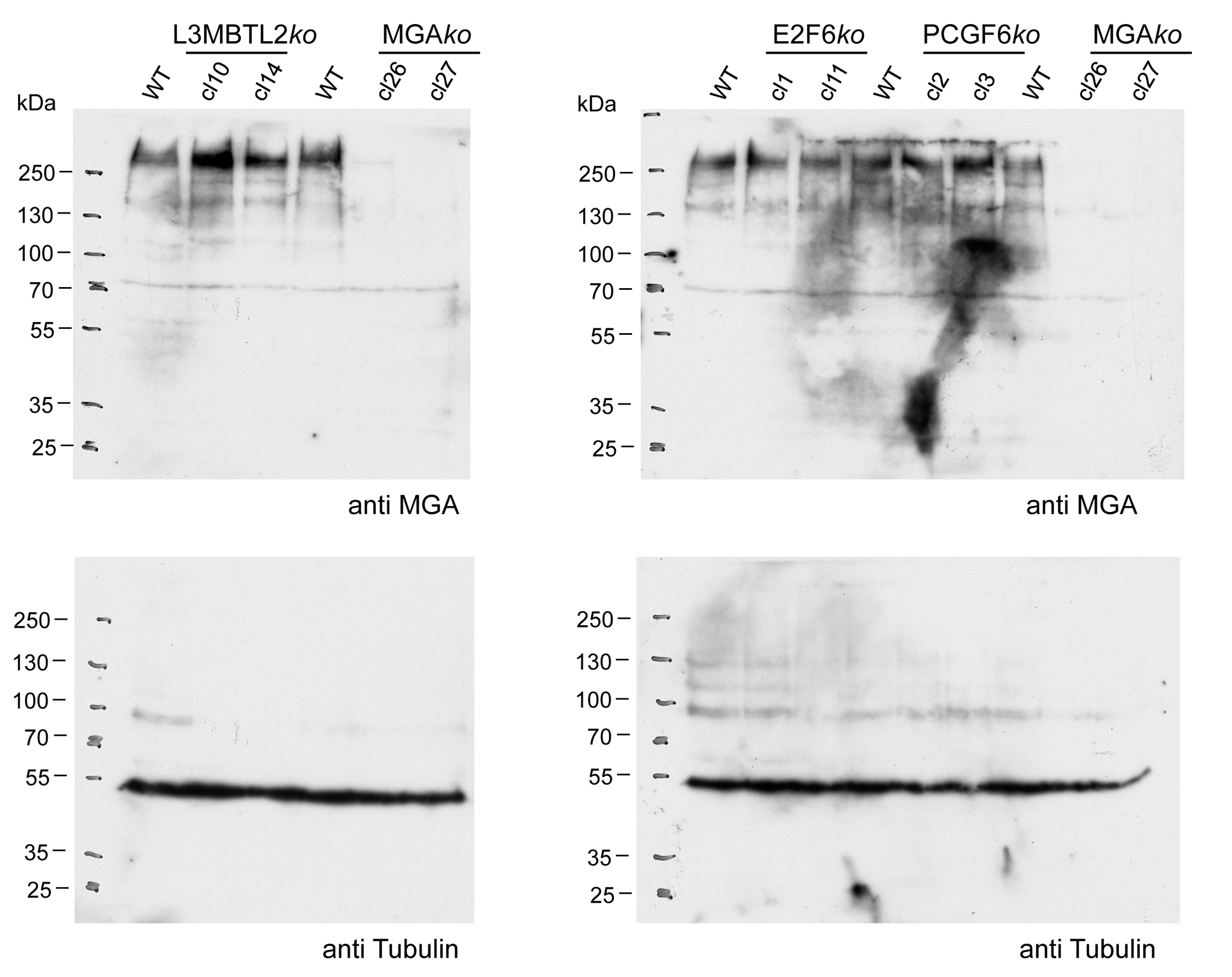

Supplement: S4 Fig — Western blot analysis of MGA with whole cell extracts from wild type (WT), MGAko, L3MBTL2ko, E2F6ko and PCGF6ko HEK293 cells. Shown are uncropped Western blots. The blots were stripped and re-probed with anti-Tubulin. (TIF) [file pgen.1007193.s004.tif]

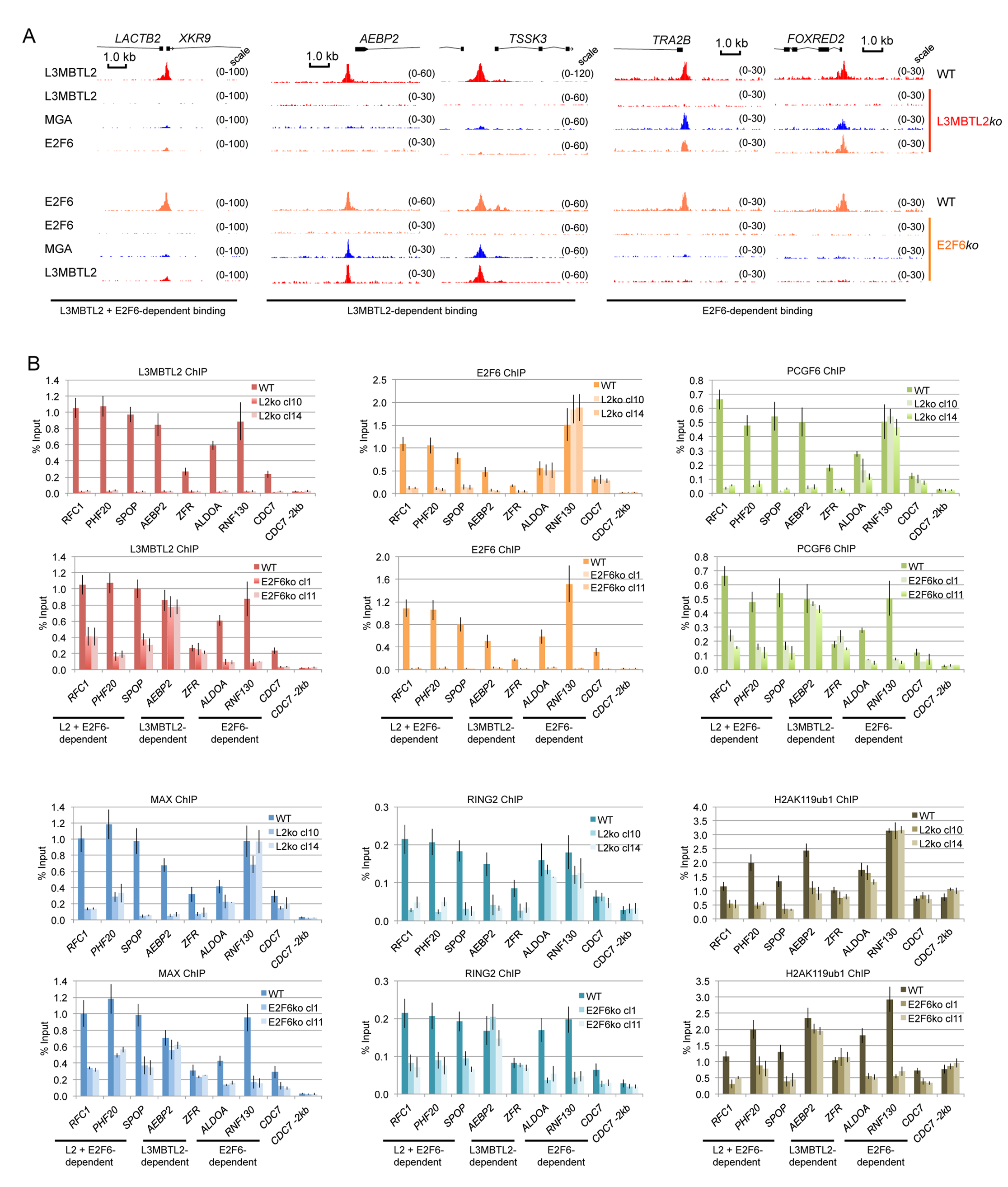

Supplement: S5 Fig — (A) Additional genome browser screenshots of ChIP-seq tracks showing differential binding of PRC1.6 components (MGA, L3MBTL2 and E2F6) in L3MBTL2ko and E2F6ko cells. Binding of MGA to the LACTB2 promoter was reduced in L3MBTL2ko and E2F6ko cells. Binding of MGA to the AEBP2 and TSSK2 promoters was lost in L3MBTL2ko cells but remained in E2F6ko cells. Conversely, binding of MGA to the TRA2B and FOXRED2 promoters was lost in E2F6ko cells but remained in L3MBTL2ko cells. (B) Local levels of L3MBTL2, E2F6, PCGF6, MAX, RING2 and H2AK119ub1 at selected PRC1.6 target promoters were determined in two different L3MBTL2ko (L2ko cl10 and L2ko cl14) and in two different E2F6ko (E2F6ko cl1 and E2F6ko cl11) cell clones by ChIP-qPCR. The CDC7 -2kb region served as a negative control region. Percent of input values represent the mean of at least three independent experiments +/- SD. (TIF) [file pgen.1007193.s005.tif]

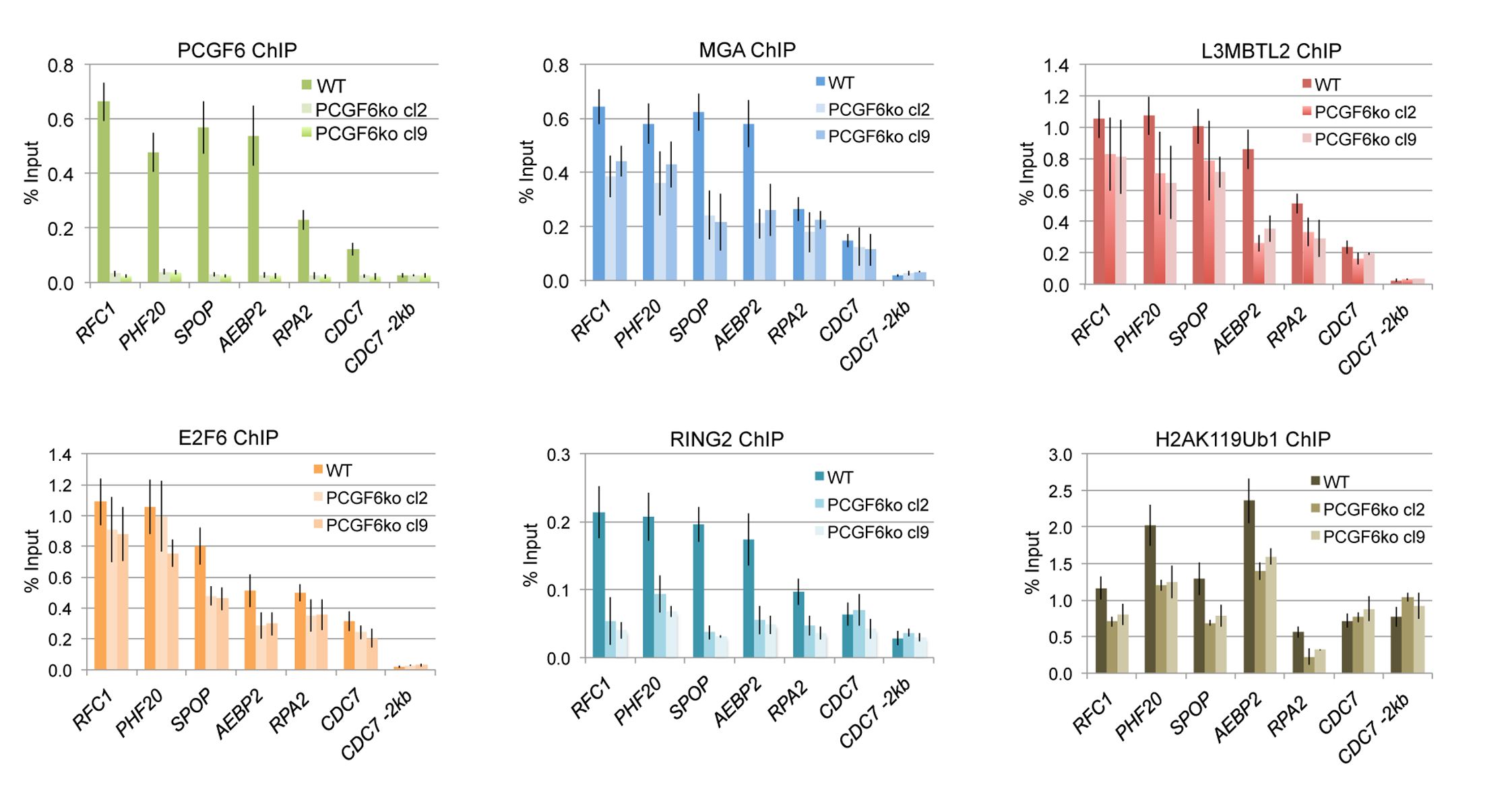

Supplement: S6 Fig — Local levels of PCGF6, MGA, L3MBTL2, E2F6, RING2 and H2AK119ub1 at selected PRC1.6 target promoters were determined in two different PCGF6ko cell clones (PCGF6ko cl2 and PCGF6ko cl9) by ChIP-qPCR. The CDC7 -2kb region served as a negative control region. Percent of input values represent the mean of at least three independent experiments +/- SD. (TIF) [file pgen.1007193.s006.tif]

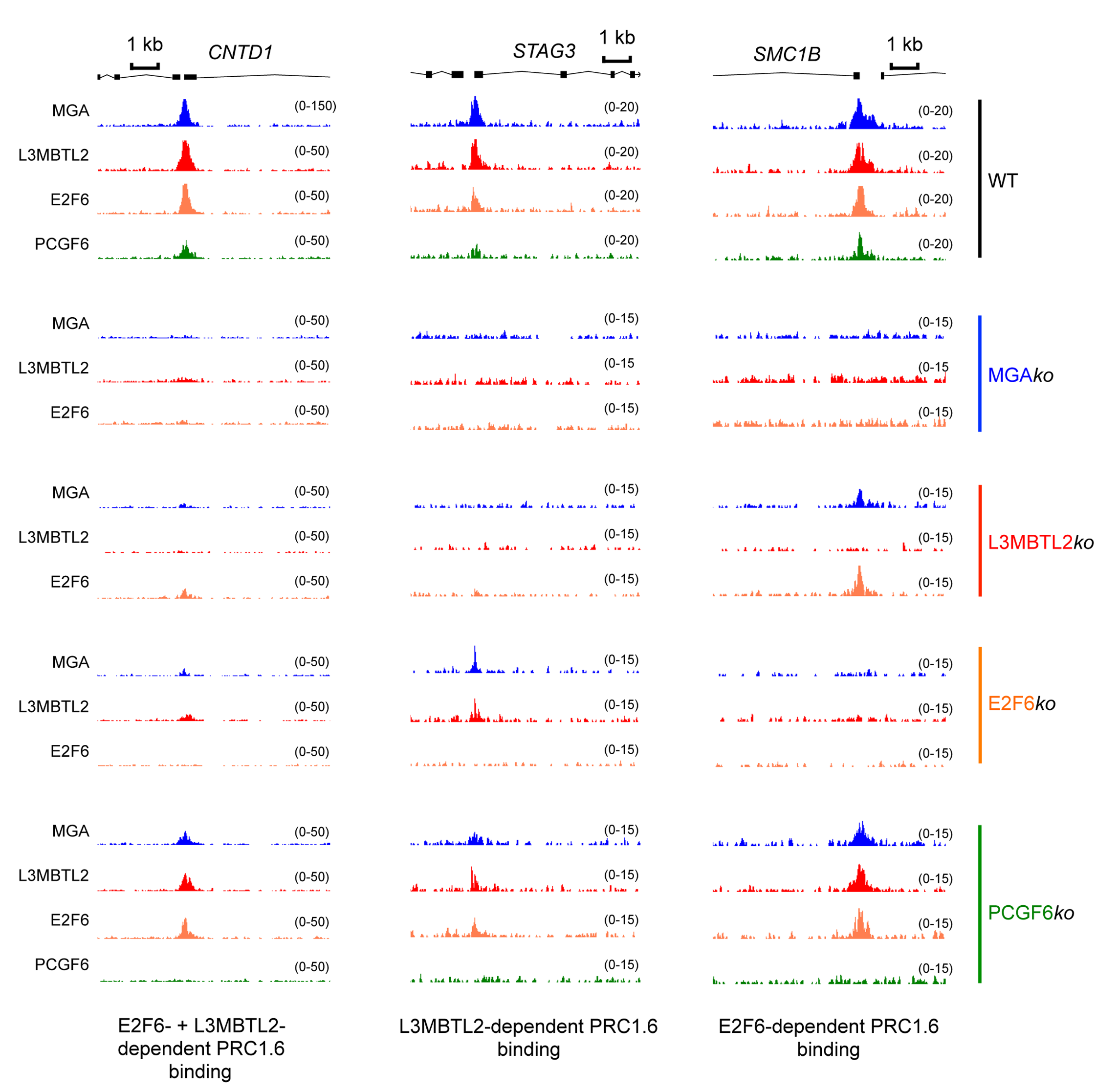

Supplement: S7 Fig — Genome browser screenshots of ChIP-seq tracks showing binding of MGA, L3MBTL2, E2F6 and PCGF6 to the CNTD1, STAG3 and SMC1B promoters in wild type cells (WT), and in MGAko, L3MBTL2ko, E2F6ko and PCGF6ko cells. (TIF) [file pgen.1007193.s007.tif]

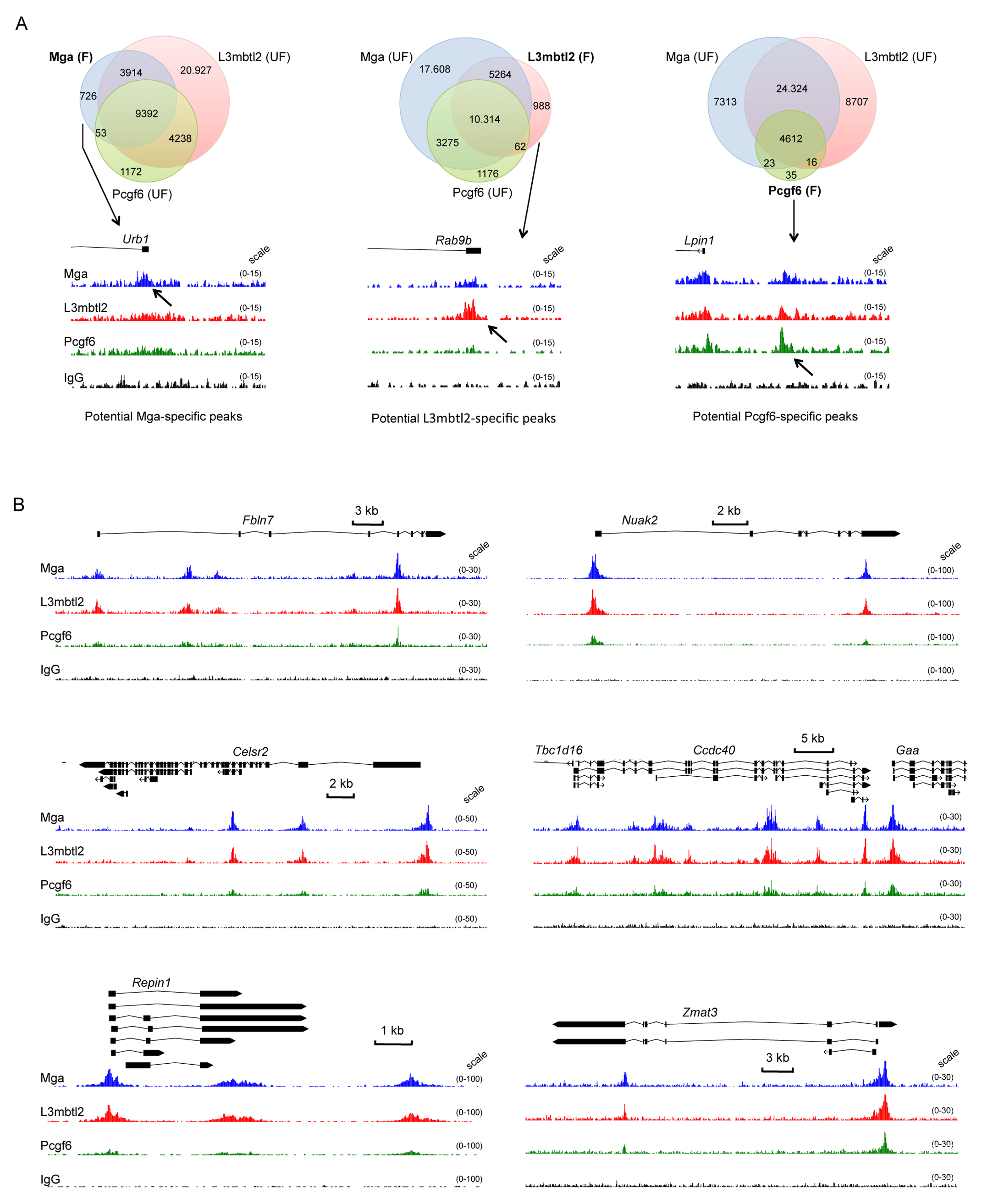

Supplement: S8 Fig — (A) Top, Venn diagrams showing the overlap of filtered Mga (left), L3mbtl2 (middle) and Pcgf6 (right) MACS peaks (F; ≥30 tags and 3x over IgG) with unfiltered MACS peaks (UF) of the two other PRC1.6 subunits. Bottom, representative genome browser screenshots of ChIP-seq tracks of potential Mga-, L3mbtl2- or E2f6-specific peaks indicate also binding the other PRC1.6 subunits. (B) Genome browser screenshots of ChIP-seq tracks showing multiple Mga, L3mbtl2 and Pcgf6 peaks in promoter regions and in gene bodies. Alternative transcripts according to Ensembl are shown above. (TIF) [file pgen.1007193.s008.tif]
